# Spaceflight Activates Lipotoxic Pathways in Mouse Liver

**DOI:** 10.1371/journal.pone.0152877

**Published:** 2016-04-20

**Authors:** Karen R. Jonscher, Alba Alfonso-Garcia, Jeffrey L. Suhalim, David J. Orlicky, Eric O. Potma, Virginia L. Ferguson, Mary L. Bouxsein, Ted A. Bateman, Louis S. Stodieck, Moshe Levi, Jacob E. Friedman, Daila S. Gridley, Michael J. Pecaut

**Affiliations:** 1 Department of Anesthesiology, University of Colorado Anschutz Medical Campus, Aurora, CO, United States of America; 2 Department of Biomedical Engineering, University of California Irvine, Irvine, CA, United States of America; 3 Beckman Laser Institute and Medical Clinic, University of California Irvine, Irvine, CA, United States of America; 4 Department of Pathology, University of Colorado Anschutz Medical Campus, Aurora, CO, United States of America; 5 Department of Mechanical Engineering, University of Colorado at Boulder, Boulder, CO, United States of America; 6 Department of Orthopedic Surgery, Harvard Medical School, Boston, MA, United States of America; 7 Department of Bioengineering, University of North Carolina at Chapel Hill, Chapel Hill, NC, United States of America; 8 BioServe Space Technologies, University of Colorado at Boulder, Boulder, CO, United States of America; 9 Department of Medicine, University of Colorado Anschutz Medical Campus, Aurora, CO, United States of America; 10 Department of Pediatrics, University of Colorado Anschutz Medical Campus, Aurora, CO, United States of America; 11 Department of Basic Sciences, Division of Radiation Research, Loma Linda University School of Medicine, Loma Linda, CA, United States of America; University of Basque Country, SPAIN

## Abstract

Spaceflight affects numerous organ systems in the body, leading to metabolic dysfunction that may have long-term consequences. Microgravity-induced alterations in liver metabolism, particularly with respect to lipids, remain largely unexplored. Here we utilize a novel systems biology approach, combining metabolomics and transcriptomics with advanced Raman microscopy, to investigate altered hepatic lipid metabolism in mice following short duration spaceflight. Mice flown aboard Space Transportation System -135, the last Shuttle mission, lose weight but redistribute lipids, particularly to the liver. Intriguingly, spaceflight mice lose retinol from lipid droplets. Both mRNA and metabolite changes suggest the retinol loss is linked to activation of PPARα-mediated pathways and potentially to hepatic stellate cell activation, both of which may be coincident with increased bile acids and early signs of liver injury. Although the 13-day flight duration is too short for frank fibrosis to develop, the retinol loss plus changes in markers of extracellular matrix remodeling raise the concern that longer duration exposure to the space environment may result in progressive liver damage, increasing the risk for nonalcoholic fatty liver disease.

## Introduction

The spaceflight environment impacts many physiological systems, resulting in potentially serious consequences, particularly for longer duration space exploration. As use of the International Space Station (ISS) is increased, and with the rise of commercial spaceflight and tourism, the systemic effects of microgravity must be carefully investigated to protect human health. Although most research has focused on bone, muscle, brain and cardiovascular function, several studies have shown that exposure to the space environment alters both energy and lipid metabolism in humans and rodents [1, 2]. In astronauts, a diabetogenic phenotype was induced, the severity of which was linked with flight duration [1, 3], suggesting the liver may be a target of spaceflight-induced deficits. Indeed, significant spaceflight-induced changes in hepatic genes linked to oxidative defense have been previously observed [4].

The liver is a major metabolic organ and the most common chronic disease worldwide affecting the liver is nonalcoholic fatty liver disease (NAFLD). NAFLD describes a spectrum of liver abnormalities, ranging from hepatic steatosis, or fat droplet accumulation, to nonalcoholic steatohepatitis (NASH), with inflammation and fibrosis causing irreversible tissue damage in many cases. NAFLD increases the risk of cardiovascular events eight-fold, type 2 diabetes mellitus three-fold, and is a strong risk factor for hepatocellular carcinoma [5]. The molecular mechanisms underlying this phenotype are not well understood and whether the liver is vulnerable to injury or dysfunction resulting from spaceflight remains an open question.

Direct cellular contact and dynamic biochemical signaling modulate crosstalk between endothelial cells, hepatic stellate cells (HSCs), Kupffer cells and hepatocytes, therefore damage to any of these cells may disrupt homeostasis and lead to NAFLD. The development of NAFLD is still poorly understood; however, several insults (“hits”) are thought to be jointly responsible for causing progressive liver injury. The “classical” hit typically involves excessive triglyceride accumulation in the hepatocytes and resultant dysfunction due to subsequent hits from oxidative stress, lipotoxicity or gut-derived bacterial endotoxin activation of Kupffer cells, inducing secretion of pro-inflammatory cytokines that activate neighboring HSCs [6, 7]. Activated HSCs then fill the Space of Disse with extracellular matrix (ECM), further diminishing hepatocyte function and setting about a positive feed-back loop that progressively diminishes hepatocyte function and nutrient availability.

The goal of this study was to more comprehensively characterize molecular and functional changes in the liver, some of which may represent early signs of NAFLD, which result from spaceflight. In liver, chronic hyperlipidemia and stress can cause activation of HSCs [8]. HSCs play an important role in the pathogenesis of liver disease and the activation of HSCs, a process involving retinoid export followed by transformation into proliferative myofibroblasts and collagen secretion, represents a final common pathway in the response of the liver to insult or injury. A characteristic of healthy HSCs is the storage of retinyl esters in multiple lipid droplets. Repeated hepatic insult due to excess lipid storage has been linked with the increasing loss of HSC lipid droplets and their retinoid stores, accompanied by progressively worsening hepatic fibrosis [9]. Notably, oxidative stress, an important and well-known component of the space environment, can activate HSCs [10], a major step in the pathway to NAFLD [11].

Utilizing a novel, multi-faceted systems biology approach and liver samples obtained from placebo-treated mice aboard Space Transportation System (STS) -135, the final 13-day US Space Shuttle mission launched on July 8, 2011, we observed changes in hepatic pathophysiology of spaceflight mice suggestive of early NAFLD. We also observed activation of a self-maintaining peroxisome proliferator-activated receptor (PPAR) alpha-driven program mediated by thioesterases, generating ω-3 fatty acids, ketone bodies, bile acids and dicarboxylic acids. Increased hepatic collagen deposition was not observed in mice subjected to this short-duration spaceflight; however, we observed profound loss of retinol and altered mRNA expression levels of several markers of HSC activation, raising the concern that a NAFLD-like phenotype that may become pathogenic might follow prolonged exposure to the space environment.

## Materials and Methods

The University of Colorado Anschutz Medical Campus Institutional Animal Care and Use Committee (IACUC) was consulted for our portion of the study and no protocol was required, since only tissues obtained after euthanasia (no live animals) were analyzed at our site. All NASA studies involving vertebrate animals are carried out in strict accordance with the recommendations in the Guide for the Care and Use of Laboratory Animals of the National Institutes of Health. The animal studies performed were reviewed and approved by multiple ACUC boards, including the NASA Ames Research Center ACUC, the NASA Kennedy Space Center (KSC) ACUC and the University of Colorado at Boulder IACUC (the home institution for the Principal Investigator of the overarching study–Dr. V. L. Ferguson), following NASA’s standard operating procedures.

### Animals and Sample Collection

Nine-week old weight-matched female C57BL/6J mice (*n* = 15/group) were placed into three Animal Enclosure Modules (AEMs, 2 groups of 5 per AEM separated by a wire mesh) and flown on the Space Shuttle Atlantis (STS-135) for approximately 13.5 days. Ground AEM controls (*n* = 15) were housed at the Space Life Science Laboratory (SLSL) at KSC. Females were selected because they produce fewer odor annoyance issues and were the only gender approved for flight, principally with the numbers of animals being flown (10 per habitat, 5 per side).

Additionally, all historical data (e.g., STS-118) were obtained on female mice so it permitted comparisons across missions, particularly for the musculoskeletal studies that were primary.

Upon receipt, all mice were provided sterilized Purina Lab Diet 5K52 (22.2% protein, 16.0% fat, 61.7% carbohydrates, caloric density 3.17 kcal/g) *ad libitum* in the food hopper and a NASA NuRFB foodbar was provided on the floor of the cage. Two days later the Purina diet was removed. Only the NASA NuRFB foodbar (2.80 kcal/g) was provided from that point forward. The NASA NuRFB powdered diet is custom ordered from Harlan Teklad (TD 04197) and is comprised of 47% carbohydrate, 17.9% protein, 3.9% lipids, 2.8% fiber, and 28% water. Long and short-term ground studies show no difference in physiological response between mice fed the NuRFB vs. the AIN-93G Growth Purified Diet recommended by the American Institute of Nutrition (18.7% protein, 16.4% fat, 65.1% carbohydrates, 3.89 kcal/g) [12, 13]. AEM control mice were placed into the same housing used in flight and environmental parameters were matched as closely as possible based on 48-hour delayed telemetry data. Conditions were controlled for temperature, humidity, and a 12:12 hr light:dark cycle; NASA foodbars and water were provided *ad libitum*. All mice were placed into AEM housing one day before flight. All mice underwent dual-energy X-ray absorptiometry (DXA) densitometry (Piximus) both pre- and post-flight. Mice were anesthetized briefly with isoflurane (< 10 minutes under anesthesia) and allowed to recover following DXA (preflight) or were immediately euthanized (postflight).

Tissues were distributed across a large team of investigators under the guidance/organization of NASA’s Biospecimen Sharing Program; we received one half lobe of liver from six mice per group. All dissections occurred at the SLSL within 3–5 h after return of the Space Shuttle Atlantis. Mice, approximately 11 weeks of age, were euthanized using 4% isoflurane followed by cardiac puncture and exsanguination. Liver lobes were extracted and dissected. A portion of the liver was prepared in 4% paraformaldehyde and the rest snap frozen in liquid nitrogen. Snap frozen and fixed livers were shipped to either Loma Linda University or University of Colorado Anschutz Medical Campus and stored appropriately prior to use. One fixed AEM liver did not provide useful sections and was not included in imaging analyses. Fat depots were dissected, weighed and flash frozen then stored at -80°C.

### Transcriptomics

A detailed description is provided in S1 File. Briefly, RNA was isolated with an RNeasy kit (Qiagen) and the Ambion WT expression kit was employed to prepare mRNA for whole transcriptome microarray analysis using an Affymetrix GeneChip 1.0ST array. Arrays were scanned using GeneChip Scanner 3000 7G and Command Console Software v. 3.2.3 to produce.CEL intensity files which were processed with CARMAweb. Results were imported into Ingenuity Pathway Analysis (Qiagen) software.

### Quantitative Real-Time PCR

RNA was isolated using an RNeasy kit as above and quantified using a NanoDrop spectrophotometer (NanoDrop Technologies). RNA was converted to cDNA using reverse transcriptase reagents (Bio-Rad Laboratories). Candidate gene expression was analyzed using the TaqMan and SYBR system based on real-time detection of accumulated fluorescence (ABI Prism Step One) as described previously [14]. Gene expression of each target sequence was normalized to expression of the endogenous control 18S rRNA. Forward and reverse sequences for primers used were as follows:

mPPARa-F: TCGCTATCCAGGCAGAAGG

mPPARa-R: AACAACAATAACCACAGA

m18s-F: TCCGATAACGAACGAGAC

m18s-R: CTAAGGGCATCACAGACC

### Total Triglyceride Analysis

Frozen liver (~ 25 mg, *n* = 6/group) was used for analysis of total triglyceride content. Lipids were extracted using 1:2:0.8 methanol:chloroform:water, dried under nitrogen, then resuspended in isopropyl alcohol with 2% Triton. Lipid extracts were mixed 1:100 with Infinity Reagent (Thermo Fisher Scientific) and triglycerides quantified using a colorimetric assay following manufacturer’s instructions.

### Metabolomics

Frozen liver pieces (*n* = 6/group) were sent to Metabolon, Inc. and stored at -80°C before use. Samples were prepared for the appropriate instrument, either LC/MS or GC/MS, as described previously [15, 16]. Briefly, automated sample preparation was conducted using a series of proprietary organic and aqueous extractions. The resulting extract, including QC standards, was divided into two fractions—one for analysis by LC and one for analysis by GC. Extracts were placed briefly on a TurboVap® (Zymark) to remove the organic solvent. Each extract was then frozen and dried under vacuum.

### Liquid Chromatography/Mass Spectrometry (LC/MS)

Extracts were split into two aliquots, dried, then reconstituted in acidic or basic LC-compatible solvents, each of which contained 11 or more injection standards at fixed concentrations. A Waters ACQUITY UPLC and a Thermo-Finnigan LTQ-FT MS were utilized for the analysis. One aliquot was analyzed using acidic positive ion optimized conditions and the other using basic negative ion optimized conditions in two independent injections using separate dedicated columns. Extracts reconstituted in acidic conditions were gradient eluted from a Waters BEH C_18_ 2.1 x 100 mm, 1.7 μm column using water and methanol both containing 0.1% formic acid, while basic extracts, which also used water/methanol, contained 6.5 mM ammonium bicarbonate. MS analysis alternated between MS and data-dependent MS^2^ scans using dynamic exclusion, scanning from 80–1000 *m/z*. Accurate mass measurements were made on precursor ions with greater than 2 million counts; typical mass error was less than 5 ppm.

### Gas Chromatography/Mass Spectrometry (GC/MS)

Extracts destined for GC/MS were re-dried under vacuum desiccation for a minimum of 24 h prior to derivatization under dried nitrogen using bis(trimethylsilyl)-trifluoroacetamide (BSTFA). The GC column was 5% phenyl/95% dimethyl polysiloxane fused silica (20 m x 0.18 mm ID; 0.18 μm film thickness) and the temperature ramp was from 40° to 300°C within 16 min; helium was used as the carrier gas. Extracts were analyzed on a Thermo-Finnigan Trace DSQ fast-scanning single-quadrupole MS using electron impact ionization set to scan from 50–750 *m/z* at unit mass resolving power.

### Compound identification

Mass spectral data were loaded into a relational database, where QC limits were imposed. Peaks were identified using Metabolon’s peak integration software [17]. Compounds were identified by comparison to library entries of purified standards or recurrent unknown entities based on the combination of chromatographic properties and mass spectra. Data normalization was performed for studies spanning multiple days to correct for variation resulting from instrument inter-day tuning differences. Each compound was corrected by registering the medians to equal 1.00 in run-day blocks and normalizing each data point proportionately. Missing values, assumed to be below the limit of detection of the instrument, were imputed with the observed minimum after normalization.

### Pathway Analysis

Ingenuity Pathway Analysis (IPA) and Exploratory Gene Association Networks (EGAN) [18] (akt.ucsf.edu/EGAN) were used to assess pathway activation based on changes in mRNA expression levels. Human Metabolite Database (HMDB) accession numbers were queried and names of genes associated with metabolites exhibiting changes in concentration were extracted manually. These genes were used to construct a subset list of genes that may be functionally significant, based on metabolomic changes, and this subset was also subjected to pathway analysis using EGAN.

### Microscopy

#### Raman spectroscopy

Coherent anti-Stokes Raman scattering (CARS) and Stimulated Raman scattering (SRS) signals were obtained by combining two laser beams: a Stokes beam fixed at 1064 nm, and a pump beam tuned to the wavelength of interest, described in detail in S1 File and S1 Table. Spectral tuning of the pump beam was possible by adjusting the crystal temperature, the Lyot filter, and the cavity length of the OPO with custom-written computer code. SRS images were obtained by detecting the stimulated Raman loss of the pump beam. For this purpose, the Stokes beam was modulated at 10 MHz with an acousto-optic modulator (AOM; Crystal Technology). Additional spontaneous Raman spectra from the mouse liver were acquired with a commercial Raman microscope (InVia Confocal; Renishaw). Images were background subtracted using Fluoview software and integrated signal intensity was quantified with ImageJ. Multiple droplets and multiple sections were analyzed from *n* = 4 mice per group.

#### Histology and immunohistochemistry

Fixed tissue sections were processed for hematoxylin and eosin (H&E) staining and immunofluorescence microscopy as described previously [19]. Additional histology sections were processed for Periodic Acid Schiff (PAS) [20] and Picrosirius Red using stains made in house (http://www.ihcworld.com/_protocols/special_stains/sirius_red.htm). Immunoreactivity for PLIN2 and ACTA2 was visualized using secondary antibodies conjugated with Alexafluor 488 or Alexafluor 594 (Life Technologies) at dilutions of 1:500 and 1:250, respectively. Nuclei were stained with 4’, 6-diamidino-2-phenylindole (DAPI; Sigma Chemical Co.). Antibody to PLIN2 was raised in rabbits as described [21] and antibody to ACTA2 was purchased from Epitomics-Abcam. Immunofluorescence images were captured on a Nikon Diaphot fluorescence microscope and digitally deconvolved using the No Neighbors algorithm (Slidebook) as described previously [19]. Histologic images were captured on an Olympus BX51 microscope equipped with a 4mp Macrofire digital camera (Optronics) using the PictureFrame Application 2.3 (Optronics). Cross-polarized light was also used to enhance visualization of Picrosirius Red stained images as previously described [22]. All images were cropped and assembled using Photoshop CS2 (Adobe Systems, Inc.).

#### Second Harmonic Generation (SHG) and Two-Photon Autofluorescence (TPAF) Microscopy

SHG and TPAF microscopy were used for label-free collagen imaging (see S1 File for details). Cryosections (16 um thick) on microscope slides were rinsed with cold phosphate-buffered saline to remove Optimal Cutting Temperature (OCT) solution and coverslipped. Sections from *n* = 3 mice per group were scanned in 2–3 regions of interest. Images were acquired at 60× and captured using Zen software (Zeiss).

### Statistical Analysis

Data were analyzed with GraphPad Prism V6.0 using a Mann-Whitney t-test or unpaired t-test with Welch’s correction for comparison between groups. Means ± SEM were reported. The ROUT method with Q = 1% was used to identify outliers for exclusion from analysis. *P* values less than 0.05 were selected to indicate significance.

## Results

### Spaceflight mice lose weight

Muscle atrophy is a well-known complication of exposure to microgravity [23]. Similar to body weight changes observed in astronauts, mice flown in space lose body mass. We subjected mice to DXA analysis, both pre- and post-flight, to determine whether spaceflight affected fat mass as well as lean mass in mice. Table 1 summarizes the DXA results, demonstrating that both groups of mice lost weight over the 13-day study period. However, FLT mice lost almost double the amount of weight compared to ground mice (12% of body weight as compared with 6% of body weight lost by AEM controls), although food intake did not significantly differ between the groups. The composition of weight loss was significantly different between groups; the FLT mice lost more lean body mass than the AEM controls, and thus exhibited increased fat as a percentage of body weight. Adipose tissue was collected and weights for inguinal, retro-peritoneal, perigonadal and brown adipose tissue (BAT) are listed in Table 1. No significant changes in fat mass were measured between FLT mice and AEM controls. Intact livers were not weighed prior to dissection and distribution, however liver mass was measured on a comparable previous flight, STS-118, where FLT mice exhibited a significant decrease in liver / brain mass as compared with AEM controls; significance was lost when liver mass was compared with body mass [4].

**Table 1:** Food intake and body composition measurements for AEM controls and FLT mice.

	AEM	FLT	FLT/AEM	*P*-value^b^
**Intake**				
Food Intake (g)^a^	4.08 ± 0.10	4.09 ± 0.18	1.00	0.865
Water Intake (g)	3.38 ± 0.22	2.73 ± 0.01	0.81	**0.038**
**Body Composition**				
ΔBody Mass (g)^c^	-1.18 ± 0.24	-2.28 ± 0.57	1.93	**0.036**
Δ% Fat^a^	4.12 ± 0.77	5.05 ± 1.84	1.22	**0.022**
**Adipose Depots**				
Inguinal (g)	0.168 ± 0.018	0.144 ± 0.024	0.859	0.447
Retro-peritoneal (g)	0.146 ± 0.022	0.147 ± 0.029	1.00	0.995
Perigonadal (g)	0.411 ± 0.054	0.433 ± 0.082	1.03	0.897
BAT (g)	0.049 ± 0.007	0.034 ± 0.004	0.682	0.092

Values represent mean ± SEM.

^a^Food and water intakes are calculated based on consumption over the 12.8-day flight for 3 cages of 5 mice in each group and are an estimate of intake per mouse per day.

^b^*P* values are obtained using a two-tailed Student’s t-test comparing AEM and FLT means.

^c^The mean change (Final–Baseline) of body mass and % fat from DXA measurements are provided; data are presented from *n* = 6 mice per group, a subset of the 15 mice/group that were originally measured, corresponding to the mice from which we received liver tissue.

**Fig 1:**
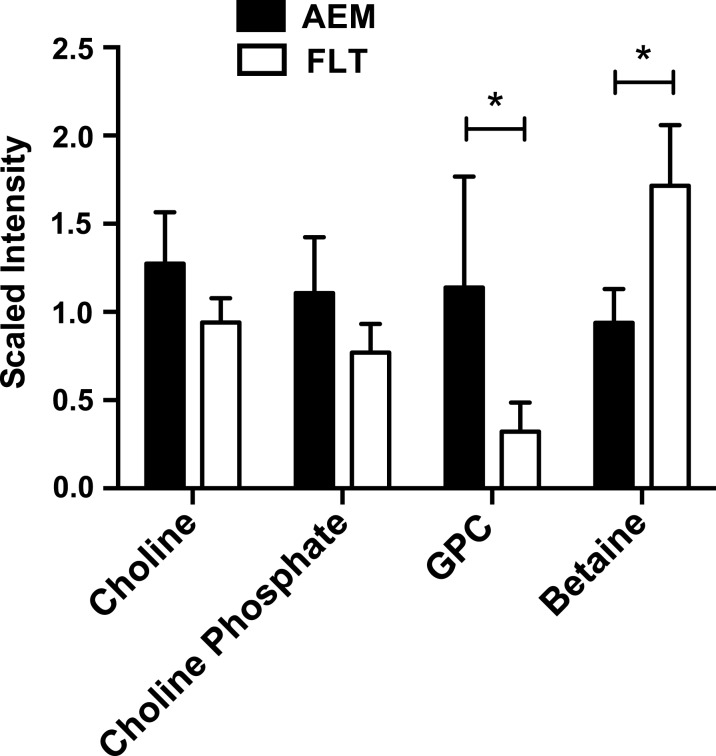
Betaine levels are increased in FLT mouse livers. Mass spectrometry-based metabolomics was performed on *n* = 6 mice per group as described in Methods and Materials. Mass spectra corresponding to choline and related metabolites were identified and extracted chromatographic peaks used for relative quantification. Mann-Whitney analysis of results showed a significant increase in levels of betaine in FLT mice as compared with AEM controls, with a concomitant decrease in concentrations of metabolic precursors. *P < 0.05; data are mean ± SEM.

Notably, FLT mice consumed nearly 20% less water than AEM controls (Table 1). In order to more comprehensively investigate effects that exposure to the space environment may have on the liver, including the response to increased concentration of amino acids arising from atrophied muscle and effects of dehydration, we performed both global transcriptomic and metabolomic analyses on liver tissue from FLT mice and AEM controls. Selected genes exhibiting significant changes in mRNA expression levels are presented in Table 2 and the complete microarray dataset is available on the NASA GeneLab site. Using untargeted metabolomics, we identified 299 biochemicals (S2 Table), 41 of which had concentrations that were either increased or decreased in livers of FLT mice with *P* values reflecting significance (< 0.05) or above (< 0.1); selected metabolites with *increased* concentrations are summarized in Table 3. Mining these data revealed a possible impact of the decreased water consumption of the FLT mice, shown in Fig 1, where concentrations of choline, choline phosphate and glycerophosphocholine are decreased, while betaine, an osmolyte produced from metabolism of choline, is increased in FLT mice. Additionally, mRNA expression of *Gpcpd1*, a glycerophosphocholine phosphodiesterase, is significantly increased in FLT mice (log_2_ FLT/AEM = 1.578, *P* = 0.0001), a potential compensatory response.

**Table 2:** mRNA expression changes for selected genes.

	Entrezid	P-value	FLT/AEM (log_2_)
**Lipid Droplets**			
*Cidec*	14311	3.82E-06	2.501
*Pnpla2*	66853	9.19E-06	1.436
*Plin5*	66968	1.13E-05	1.022
*Plin4*	57435	2.30E-03	0.932
*Plin2*	11520	8.61E-05	0.782
*Plin3*	66905	6.47E-04	0.444
**Stellate Cell Activation**			
*Lamb3*	16780	1.17E-04	0.972
*Lepr*	16847	1.58E-02	0.787
*Serpine1*	18787	3.58E-03	0.442
*Tgfbr3*	21814	7.69E-04	0.423
*Tgfbr2*	21813	3.54E-03	0.39
*Tgfbr1*	21812	8.26E-03	0.362
*Col3a1*	12825	2.94E-02	-0.179
*Lama4*	16775	2.95E-02	-0.193
*Mmp2*	17390	3.46E-04	-0.21
*Col1a1*	12842	1.52E-02	-0.221
*Mmp19*	58223	3.07E-04	-0.383
*Vim*	22352	3.65E-01	-0.126
*Des*	13346	3.82E-01	-0.122
*Acta2*	11475	2.24E-01	-0.238
**Thioesterases**			
*Acot2*	171210	1.15E-05	2.055
*Acot1*	26897	5.06E-06	1.357
*Acot5*	217698	1.30E-04	1.189
*Acot8*	170789	1.24E-06	0.856
**Ketogenesis**			
*Bdh1*	71911	5.95E-04	1.008
*Acat1*	110446	7.53E-05	0.474
*Hmgcl*	15356	8.32E-05	0.466
*Hadha*	97212	6.43E-05	0.314
**Triglyceride Biosynthesis**			
*Agpat9*	231510	3.20E-06	1.992
*Elovl3*	12686	4.11E-05	1.865
*Lclat1*	225010	8.00E-04	0.911
*Ppp2r2d*	52432	3.49E-05	0.814
*Agpat6*	102247	7.65E-04	0.508
*Elovl1*	54325	1.10E-03	-0.473
*Elovl6*	170439	3.09E-05	-1.308
**Retinol Metabolism**			
*Cyp4a31*	666168	7.03E-05	1.427
*Retsat*	67442	7.85E-06	1.049
*Hsd17b6*	27400	2.47E-02	0.702
*Bcmo1*	63857	9.18E-03	0.565
*Cyp4a32*	100040843	2.47E-02	0.514
*Lrat*	79235	4.68E-03	-0.383
*Cyp2c68*	433247	4.47E-03	-0.513
*Dhrs3*	20148	2.18E-03	-0.521
*Cyp2c38*	13097	1.97E-02	-0.551
*Cyp2c70*	226105	6.68E-04	-0.568
*Cyp26a1*	13082	3.06E-03	-1.114
**PPARα targets**			
*Gpd2*	14571	6.20E-07	1.476
*Fgf21*	56636	1.84E-05	1.018
*Cd36*	12491	1.82E-03	0.868
*Slc27A1*	26457	2.41E-04	0.673
*Ppargc1a*	19017	5.69E-02	0.467
*Gyk*	14933	9.29E-06	0.512
**FXR targets and bile acid metabolism**			
*Fgf21*	56636	1.84E-05	1.018
*Slc22a7*	108114	9.75E-03	0.735
*Abcg5*	27409	2.89E-03	0.614
*Baat*	12012	8.11E-04	-0.284
*Hmgcr*	15357	2.62E-02	-0.525
**Miscellaneous**			
*Pparg*	19016	4.05E-03	0.965
*Cpt1a*	12894	8.26E-06	0.8
*Ucp2*	22228	4.53E-02	0.471

Fold changes, *P* values are calculated using CARMAWeb. *P* < 0.05 is considered significant. The Entrez accession number is given by Entrezid.

**Table 3:** Hepatic metabolites with increasing concentrations in spaceflight.

Biochemical Name	FLT/AEM (log2)	P-value	Sub Pathway
glutarate (pentanedioate)	2.25	0.0001	Lysine metabolism
1-linoleoylglycerol (1-monolinolein)	1.96	0.0562	Monoacylglycerol
3-methylglutarylcarnitine (C6)	1.8	0.0006	Valine, leucine and isoleucine metabolism
3-hydroxybutyrate (BHBA)	1.69	0.0001	Ketone bodies
taurodeoxycholate	1.58	0.05	Bile acid metabolism
cholate	1.38	0.0498	Bile acid metabolism
hydroxyisovaleroyl carnitine	1.36	0.0137	Valine, leucine and isoleucine metabolism
propionylcarnitine	1.29	0.0002	Fatty acid metabolism (also BCAA metabolism)
ophthalmate	1.22	0.013	Glutathione metabolism
dimethylglycine	1.13	0.0019	Glycine, serine and threonine metabolism
hexadecanedioate	1.13	0.0021	Fatty acid, dicarboxylate
stearidonate (18:4n3)	0.99	0.0275	Long chain fatty acid
betaine	0.87	0.0009	Glycine, serine and threonine metabolism
docosapentaenoate (n3 DPA; 22:5n3)	0.86	0.0085	Essential fatty acid
uracil	0.83	0.0175	Pyrimidine metabolism, uracil containing
tetradecanedioate	0.8	0.0043	Fatty acid, dicarboxylate
oleate (18:1n9)	0.77	0.0194	Long chain fatty acid
2-aminobutyrate	0.7	0.0371	Butanoate metabolism
myristoleate (14:1n5)	0.62	0.0482	Long chain fatty acid
linoleate (18:2n6)	0.6	0.0174	Essential fatty acid
corticosterone	0.6	0.0088	Sterol/Steroid
glycerol	0.51	0.035	Glycerolipid metabolism
docosahexaenoate (DHA; 22:6n3)	0.5	0.0435	Essential fatty acid
carnitine	0.48	0.0004	Carnitine metabolism

*P* < 0.05 is considered significant. *P* values calculated using t-test with Welch’s correction.

### Spaceflight mice accumulate hepatic lipid droplets

**Fig 2:**
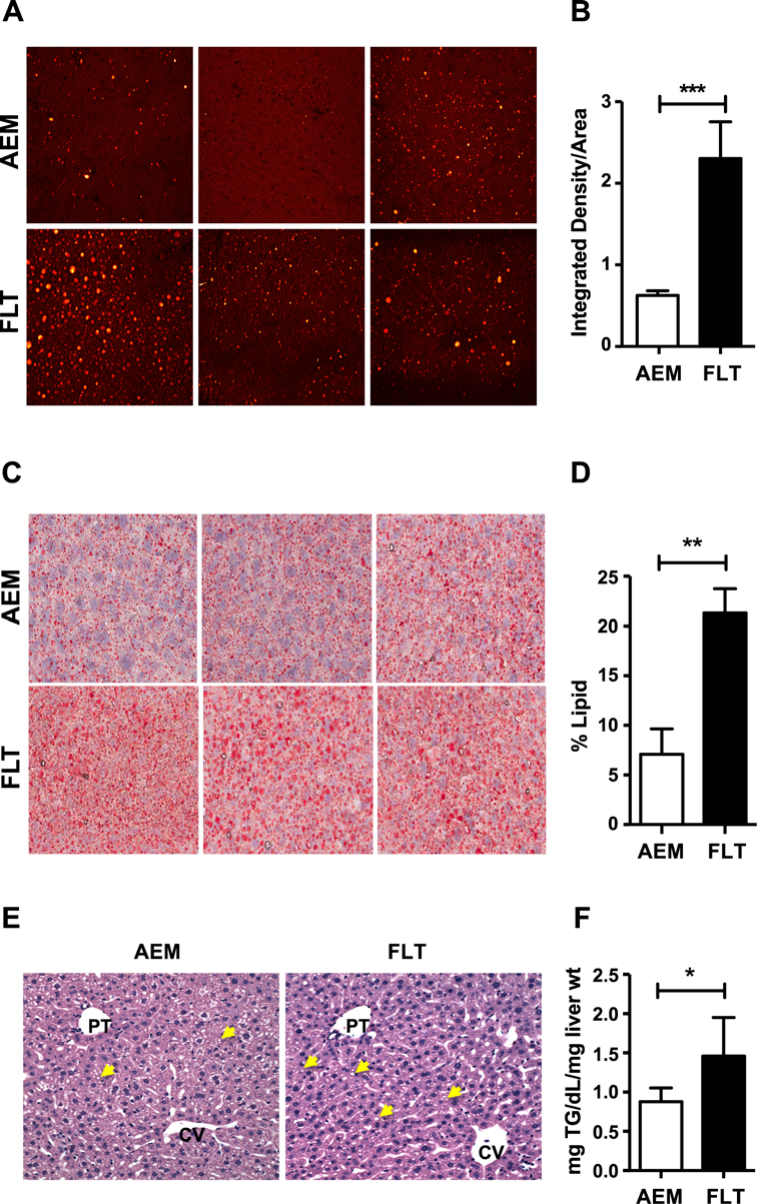
Spaceflight mice have increased accumulation of hepatic lipid droplets. A) Frozen liver tissue was cryosectioned using OCT solution and sections imaged by CARS at a magnification of 60×. Representative images are shown from 3 different animals in each group. Images from AEM ground controls appear on the top panel and FLT mice on the bottom panel. B) Multiple regions were imaged from 2 cryosections taken at different tissue depths per animal (*n* = 5/group). Images were processed using ImageJ and integrated pixel intensity measured for each unit area. A Mann-Whitney test was used to compare integrated intensity values between groups. ***P < 0.001. C) Cryosectioned samples were stained with Oil Red O and imaged at 40× using an Aperio scanner. Sections from AEM and FLT mice were analyzed. Representative images from 3 mice per group are shown. D) Following color deconvolution, where dark red staining was used as an indicator of positive lipid signal, the percent of positive lipid signal was calculated per unit area for samples from *n* = 5 mice per group. A Mann-Whitney test was used to compare between groups and the mean ± SEM is shown. ** *P* < 0.01. E) H&E staining was performed on fixed liver sections from *n* = 4–5 mice/group to investigate liver histology. Inspection of the H&E stained sections revealed that the AEM ground control mice (left panel) had predominantly small zone 2 cytoplasmic lipid droplets (CLD) whereas the FLT mice (right panel) had an increase in slightly larger CLD in a panlobular pattern. Multiple lipid droplets are indicated using yellow arrows in representative images from each group. F) Total triglycerides were measured from *n* = 6 mice/group using a colorimetric assay (540 nm). A Mann-Whitney test was used to compare the calculated concentration values between groups. Data shown are mean ± SEM. * *P* < 0.05.

The liver is a key target for metabolic processing of excess lipids. Our measurements of increased % body fat and decreased hepatic choline led us to investigate whether exposure to space would target fat accretion in the liver. We therefore used CARS microscopy to image lipid droplets in livers of AEM controls and FLT mice. CARS is used to visualize and identify lipids by tuning into resonance with the strong Raman-active vibrational mode at 2845 cm^-1^, corresponding to the symmetric stretching vibration of the CH_2_ portion of fatty acids [24]. As shown in Fig 2*A*, and consistent with what we additionally quantified by Oil Red O staining (2*C*) and observed using H&E staining (2*E*), liver cryosections from FLT mice showed significant accumulation of hepatic fat (lower panels) whereas those from AEM controls had very few lipid droplets (upper panels). Quantification of signal intensity from 2–3 regions and two sections from *n* = 5 mice per group revealed a ~3.5-fold increase in signal from lipid droplets in flight mice as compared with AEM controls (2*B* and *D*). Total triglycerides were also significantly increased in FLT mice as compared with AEM controls (2*F*).

We next interrogated the multi-‘omics datasets to determine whether triglyceride biosynthetic pathways are activated by spaceflight, leading to the observed increase in hepatic lipid storage. Indeed, we found upregulated expression levels of several genes involved in triglyceride biosynthesis (Table 2). Additionally, expression levels of *Plin2*, a marker for hepatic steatosis, as well as *Plin3* and *Plin4*, genes thought to mediate formation of nascent droplets [25], were also significantly elevated, suggesting increased synthesis and packaging of triglycerides. However, expression of hepatic carnitine palmitoyltransferase-1a (*Cpt1a*), an enzyme involved in transporting fatty acids into the mitochondria for β-oxidation, was concomitantly upregulated and the concentration of glycerol in FLT mice was augmented (log_2_ FLT/AEM = 0.51, *P* = 0.035; Table 3). Markers of triglyceride lipolysis and lipid droplet metabolism, such as *Pnpla2* (*Atgl*), *Plin5* and *Cidec* were similarly upregulated in FLT mice. As noted above, concentrations of choline, choline phosphate and glycerophosphocholine are decreased in FLT as compared with AEM (Fig 1, S2 Table), perhaps contributing membrane material to surround new or larger triglyceride-packed lipid droplets. Given that total triglycerides are elevated (2*F*), these data suggest that spaceflight shifts the balance between lipid synthesis and oxidation. The end result is increased storage, which we suggest is a protective response to an excess of toxic lipids overloading mitochondrial oxidative capacity.

### Retinoids are lost from spaceflight mouse lipid droplets

**Fig 3:**
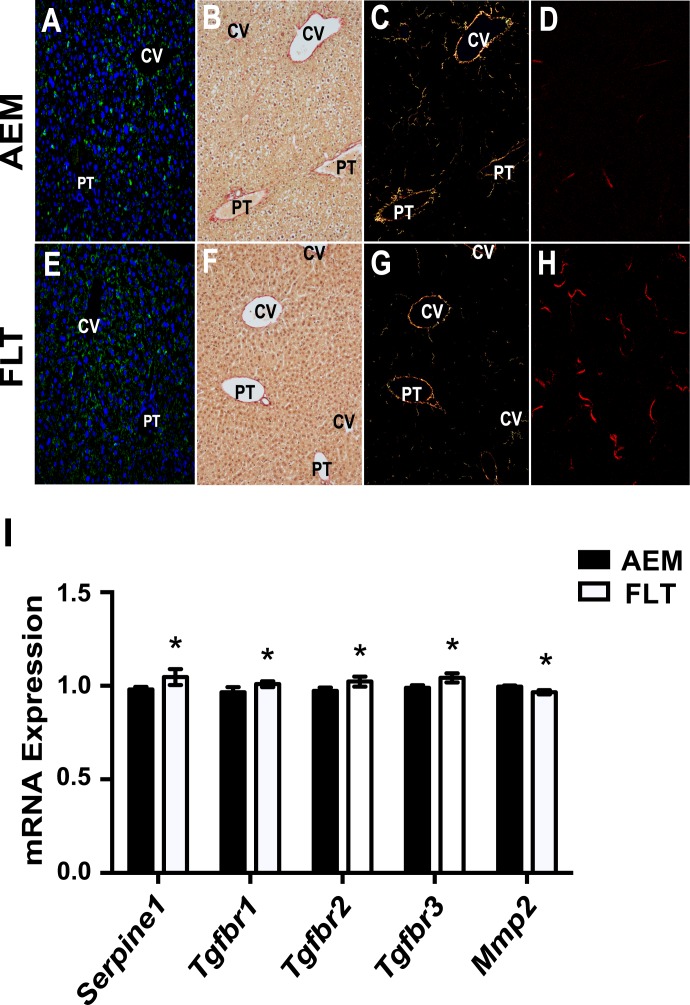
Lipid droplets in spaceflight mouse liver have reduced retinol content. Stimulated Raman scattering (SRS) images of liver sections (left panel) revealed a decreased intensity of embedded lipid droplets at ~1593 cm^-1^ for spaceflight mice (FLT, *C*) with respect to ground mice (AEM, *A*), and showed no difference at 2845 cm^-1^ (*B*, *D*), the C-H_2_ symmetric stretching band characteristic of lipids. Hyperspectral SRS imaging (right panel) around the two frequencies of interest unveil quasi-identical spectra of the lipid droplets of the two mouse groups, except for the peak at ~1593 cm^-1^ (red curves). Spontaneous Raman spectra (black curves) agree with the SRS results. Raman spectra were acquired using *n* = 3 mice per group, 2 sections per mouse, and 2–3 droplets per section. Technical replicates were also performed for several samples. SRS imaging was used to confirm the Raman results and was performed on sections from one mouse in each group.

The increased hepatic fat in FLT mice observed using CARS and Oil Red O staining led us to question whether the lipid composition of the droplets in the FLT mice differed from those in the AEM controls. We first used Raman spectroscopy to probe individual droplets. Displayed in Fig 3 are hyperspectral stimulated Raman scattering (SRS) images excited around the retinoid band (1593 cm^-1^) or the lipid band (2845 cm^-1^) (left panel) and spontaneous confocal Raman spectra acquired over a broad spectral range (right panel). Spontaneous confocal Raman spectra from small lipid droplets did not differ between the two groups; therefore, we focused on larger droplets that were highly prominent in the FLT mice. The SRS images showed a dramatic drop of the retinoid signal near ~1593 cm^-1^ for the FLT mice (3*C*), which was not observed in the AEM control mice (3*A*) when both liver sections were excited at the same frequency. By contrast, when imaged at the lipid band of 2845cm^-1^, liver sections of both AEM (3*B*) and FLT (3*D*) mice displayed intense and well-defined lipid droplets suggesting that the lipid composition of droplets in FLT mice differed primarily in the levels of retinoids present within the droplets. These data are recapitulated in the SRS spectra (red curves) plotted in the panel on the right. Except for the peak at ~1593 cm^-1^, the SRS spectra are remarkably similar for the two groups: in the vicinity of the 1593 cm^-1^ peak, both spectra showed the C = C stretching mode at 1660 cm^-1^, characteristic of unsaturated lipids. The C-H stretching band (2800–3050 cm^-1^) displayed almost identical signatures for both groups. Additional spontaneous confocal Raman spectra (black curves) confirm the similarities throughout the entire spectral range examined (from 1400 to 3100 cm^-1^). A measurement of lipid unsaturation (ratio I1660/ I1445) was not significantly different between the groups. The notable difference between the Raman spectra was the lack of a peak at 1593 cm^-1^ in the spaceflight mice, which closely matches the spectral differences seen in the hyperspectral SRS images. The peak at 1593 cm^-1^ was attributed to the conjugated C = C stretch vibrations of retinol by comparison with a pure standard (S1 Fig). The relevant vibrational modes and their molecular assignments [26, 27] are detailed in S3 Table.

### Expression levels of several HSC activation markers are altered in spaceflight

**Fig 4:**
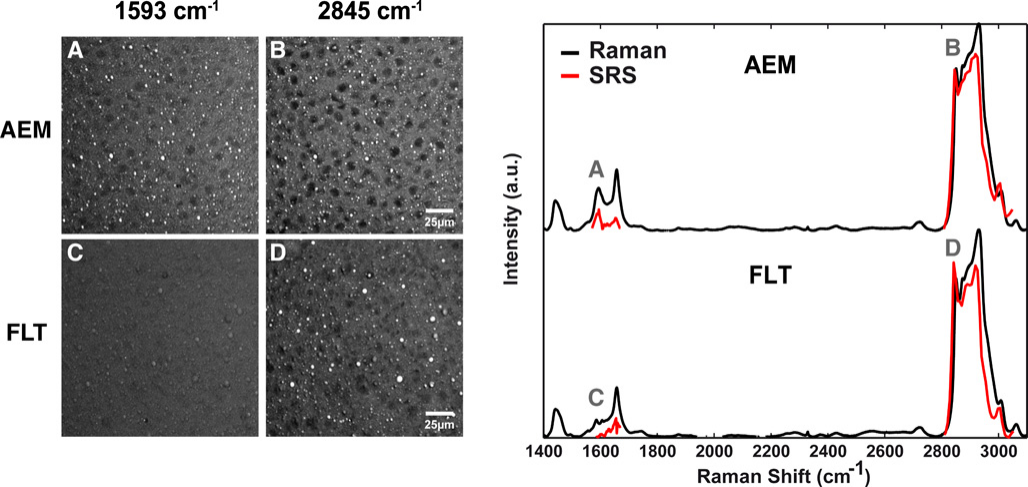
Remodeling of the ECM is increased in livers from spaceflight mice. Fixed tissues were stained for PLIN2 (green) and ACTA2 (red) and nuclei were stained with DAPI (blue) to visualize lipid droplets and markers of activation in stellate cells (*A*, *E*). Mouse liver tissues were either fixed as described in Materials and Methods and stained with Picrosirius Red (*B*, *C*, *F*, *G*) or flash frozen and cryosectioned (*D*, *H*). Collagen staining was observed in Picrosirius Red stained sections (*B*, *F*) primarily near the portal triad (PT) and central vein (CV) areas. Using cross-polarized light, signal was more readily observed (*C*, *G*). SHG imaging was performed on cryosectioned samples (*D*, *H*) and increased collagen signal in interstitial regions was observed in livers from FLT mice as compared with AEM controls. Representative images are shown from each group (*n* = 3–5 mice/group). Magnification is 200× for stained samples and 60× for SHG images. Transcriptomic analysis reveals that expression levels of several important modulators of ECM remodeling are changed in spaceflight (*I*). Values are mean ± SEM; * P < 0.05. *n* = 5–6 / group.

Retinoids are primarily stored in HSCs and are exported during activation. Since we observed an apparent export of retinoids from FLT lipid droplets, we wanted to determine if exposure to space initiated HSC activation. We first immunohistochemically stained fixed liver tissues for the presence of PLIN2, a marker for non-activated stellate cells (Fig 4A and 4E) [28]. Inspection of the sections at 200× magnification revealed PLIN2 staining primarily on lipid droplets contained within HSC cells; AEM control mice appeared to have more abundant large, non-membrane bound droplets than livers from FLT mice. We also counted the number of HSCs containing lipid droplets (non-activated HSC cells) in PLIN2-stained sections from 4 AEM and 4 FLT mice and found a slight decrease in the FLT animals that failed to reach significance (FLT/AEM = 0.81, *P* = 0.22). *Lrat* expression, required for retinyl ester synthesis and HSC lipid droplet formation, decreased in FLT mice livers as well (Table 2). To examine activation at the protein level, we immunohistochemically stained fixed tissues for the presence of alpha-smooth muscle actin (ACTA2; α-SMA), the canonical marker for activated stellate cells, and did not observe increased ACTA2 signal with spaceflight (Fig 4A and 4E; shown in red); *Acta2* mRNA expression was also not increased (Table 2). There was no difference in expression of HSC markers vimentin (*Vim*) and desmin (*Des*) between the groups suggesting HSCs were not decreased in FLT mice. We also observed a significant increase in expression of the leptin receptor, *Lepr*, which when stimulated can promote the myofibroblastic HSC transition. Taken together, these data suggest there may be fewer non-activated HSC cells in the FLT mice livers than in the AEM controls; the transition to a fibrogenic phenotype may be initiated for a fraction of the population.

Formation of a fibrotic scar requires both remodeling of the ECM and secretion/organization of collagen fibrils. To determine whether fibrosis was occurring, we quantified mRNA expression levels of a number of collagen genes and found they were either not significantly changed or were slightly downregulated. We additionally stained fixed tissues from *n* = 4 mice per group with Picrosirius Red (Fig 4B and 4F; PS Red) to visualize collagen fibrils. When viewed with cross-polarized light, the birefringence of the dye bound to the collagen fibers becomes more apparent, as seen in Fig 4C and 4G. The presence of collagen is most abundant around central veins (CV) and portal triads (PT), as expected, however fibrils are not readily observed in other regions of the tissue. Quantification of signal from the birefringent images did not yield significant differences between the groups (data not shown). We imaged cryosectioned tissues using label-free SHG microscopy. This technique allows for direct visualization of collagen (particularly fibrillary type I collagen, which tends to generate larger fibers, compared with collagen type III, which forms fine fibrils) in unstained tissue. Fig 4*H* suggests that signal from collagen fibers is increased primarily in the sinusoidal regions of FLT livers as compared with the AEM controls (4*D*). Although collagen formation was not remarkable, transcript expression levels for several markers for remodeling of the ECM were altered (Table 2, Fig 4*I*). In particular, mRNA expression levels of *Serpine1* (PAI-1), a hepatic pro-fibrotic marker [29], and TGF-β receptor subunits (*Tgfbr*) 1, 2 and 3 were significantly upregulated, while that of *Mmp2* was decreased. Taken together, these data do not demonstrate the presence of pathological fibrosis, which would be unlikely following only two weeks of spaceflight. However, they do show a pattern of upregulation of markers for activation and remodeling of the ECM that may be indicators for nascent fibrinogenic activity.

### Metabolomic and transcriptomic changes in spaceflight mouse livers suggest activation of PPARα-directed pathways

**Fig 5:**
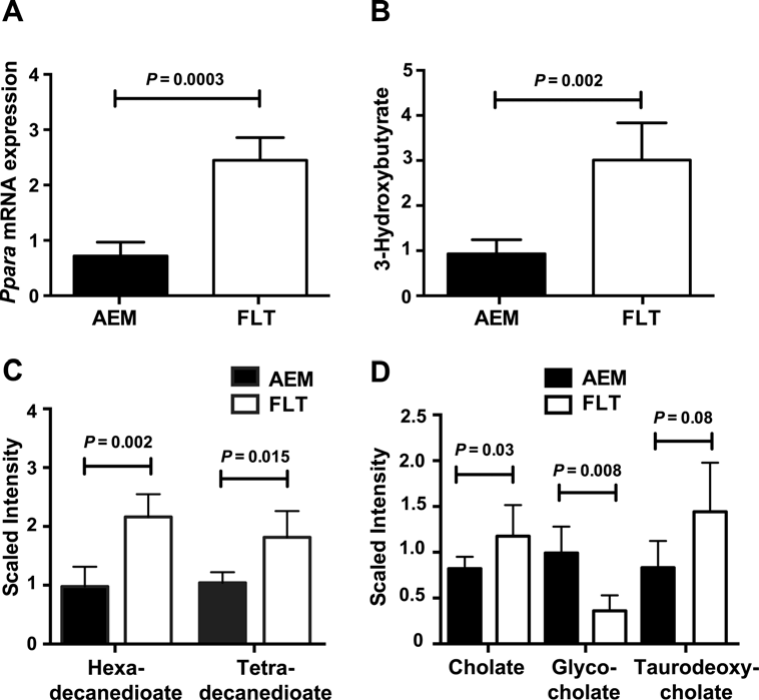
Products of PPARα-mediated thioesterase activity are upregulated in spaceflight mice. *A*) Hepatic PPARα mRNA expression was quantified using qRT-PCR and normalized to 18S rRNA levels for both FLT mice and AEM controls. An unpaired t-test with Welch’s correction was used for comparison. PPARα targets include acyl-coA thioesterases, which act in different cellular compartments to mediate lipid metabolism. Mass spectrometry-based metabolomics analysis of liver compounds showed increased concentrations of products of *B*) ACOT2, *C*) ACOT1 and *D*) ACOT8 activity. Data (mean ± SEM) were plotted for *n* = 3–5 mice/group.

Retinoids released from activated HSC droplets may be transported by retinol-binding protein through the bloodstream and taken up by other cells or may act locally. Together with released fatty acids, retinoids activate transcriptional programs mediated by nuclear receptors important in lipid metabolism, such as PPAR and RXR [30]. In order to elucidate potential transcriptional programs that may be affected by exposure to space, we used the subset of significantly increased metabolites (Table 3) and extracted genes associated with those metabolites from the HMDB, then performed an enrichment analysis on that gene subset using EGAN. S4 Table summarizes the top 50 most enriched KEGG pathways and GO process terms associated with the gene subset corresponding to metabolites with *increased* concentration in FLT livers. The majority of genes mapped to metabolic pathways, in particular to fat digestion and absorption. In addition to fatty acid metabolism, PPAR signaling was also a highly enriched pathway, involving ~20% of the genes in the subset. We measured the mRNA expression of *Ppara* by qRT-PCR and found a significant increase in livers of FLT mice (Fig 5*A*). We then performed a functional analysis of PPAR pathways using IPA including the entire transcriptomic dataset, and genes regulated by PPARα were preferentially activated (Z score = 6.196); indeed, PPARα was the most significant of the upstream regulators identified. IPA and EGAN analysis of enrichment of genes with relevant KEGG terms (complete gene set: 72/81 genes observed, *P* = 6.4 E-29; 10 genes associated with upregulated metabolites in S4 Table, *P* = 7.48 E-15) shows spaceflight resulted in preferential activation of the PPARα-mediated pathway. We additionally mapped metabolomics data onto the PPARα pathway. Biomart and GO terms obtained from AMIGO2 [31] were used to generate lists of genes related to several of these pathways. Expression of the PPARα activator PGC1-α (*Ppargc1a*) was increased and an IPA Upstream Transcription Regulator analysis also showed activation of PGC1-α mediated pathways (Z = 3.95). Expression levels of *Retsat* and *Fgf21*, other notable PPARα targets were increased, however expression of HNF4α, another important regulator of hepatic fat metabolism, was unchanged and IPA analysis did not show significant upregulation of HNF4α-modulated pathways. Our data demonstrate a remarkable pattern of increased expression of PPARα-induced genes important in fatty acid uptake (*Cd36* and *Slc27a1* [*Fatp*]), glycerol metabolism (*Gpd2* and *Gyk*) and thioesterase activity (ACOTs) correlating with spaceflight. Pathways downstream of PPARγ, ROR and RAR did not appear to be activated.

ACOTs are highly regulated by PPARα and we observed increased mRNA expression levels of *Acot 1*, *2*, *5* and *8* (Table 2). We therefore focused on mining the transcriptomics and metabolomics data to elucidate the effect of spaceflight-induced modulation of PPARα on thioesterase function. In the mitochondria, ACOT2 hydrolyzes fatty acids and has been implicated in the generation of ketone bodies following stimulation with fenofibrate, a PPARα agonist [32]. We observed a significant elevation in the concentration of the ketone body β-hydroxybutyrate (Fig 5*B*, Table 3). Only a slight increase in mRNA expression of ketogenesis genes was observed in FLT mice and *Ucp2*, a mitochondrial uncoupling protein, was upregulated (Table 2). In the cytosol, ACOT1 is thought to play a role in ω-oxidation of fatty acids by CYP4A enzymes activated by PPARα. We observed a significant increase in the concentration of the dicarboxylic acids, tetradecanedioate and hexadecanedioate, products of ω-oxidation (Fig 5*C*, Table 3).

Glutaryl-coA is a substrate for ACOT8 and elevated glutarate was detected in FLT mice (Table 3). In the peroxisome, ACOT8 regulates secretion of free and conjugated bile acids. We observed a significant FLT-related increase in the concentration of cholate (Fig 5*D*, Table 3) whereas *Hmgcr* expression was downregulated (Table 2) and precursor hepatic cholesterol levels were unchanged (S2 Table). Expression levels of *Abcg5/8*, key genes involved in the export of cholesterol and biliary sterols, are upregulated; however, expression of the bile salt export pump *Abcb11* was decreased and the uptake transporter *Slc22a7* increased (Table 2). The concentration of glycocholate was significantly decreased (Fig 5*D*, Table 3), concomitantly with downregulated expression of *Baat*, a gene responsible for glycine conjugation of bile acids (Table 2). Taken together, these data suggest spaceflight modulates cholesterol and bile acid metabolism, resulting in a net accumulation of deconjugated bile acids in the liver. We did not have access to blood to measure serum cholesterol levels or bile acids, nor did we have access to intestines to measure intestinal bile acids, therefore our data are limited and only reflect changes in hepatic bile acid pools. Although *Shp and Cyp7A1* mRNA expression levels were not elevated, pathway analysis showed activation of some FXR-mediated pathways (S2 Fig) and mRNA expression of *Fgf21*, a target of both PPARα and FXR, was highly upregulated in FLT mice (Table 2). Taken together, these data demonstrate a spaceflight-induced increase in activity of PPARα-driven pathways, particularly those mediated by ACOTs.

**Fig 6:**
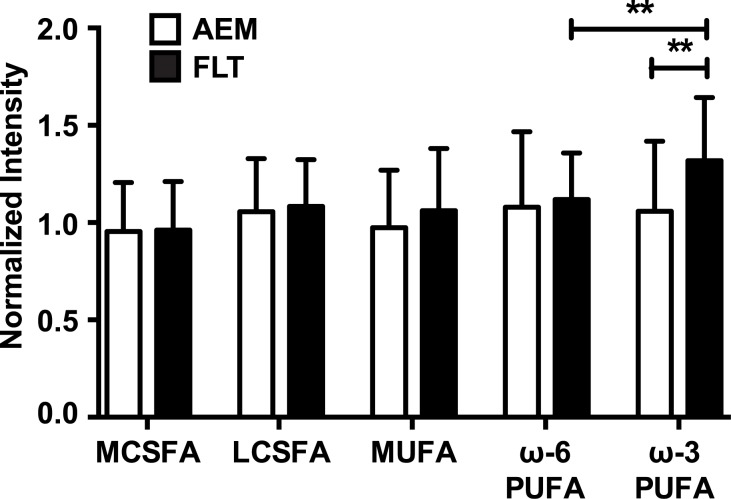
Increased ω-3 polyunsaturated fatty acid concentration following spaceflight. Mass spectrometry-based metabolomics was performed and spectra corresponding to both saturated and unsaturated fatty acids were identified and chromatographic peaks quantified. Mann-Whitney analysis of results from *n* = 5 mice per group showed a significant increase in levels of ω-3 PUFAs in spaceflight mice as compared with AEM controls. **P < 0.01; data are mean ± SEM.

Fatty acids are natural ligands for PPARs, therefore fatty acid profiles from the metabolomics data were compared between AEM and FLT mice to investigate potential activators of PPARα signaling. Fig 6 summarizes concentrations of fatty acids obtained using mass spectrometry, grouped into medium and long chain saturated fatty acids (MCSFA, LCSFA), monounsaturated fatty acids (MUFA) and ω-6 and ω-3 polyunsaturated fatty acids (ω-6/ω-3-PUFA). Heat maps show the relative concentrations of the individual fatty acids for both AEM and FLT (*n* = 5/group), plotted in S3 Fig. One-way ANOVA test with multiple comparisons between the five groups for AEM and FLT revealed that the concentrations of ω-3 fatty acids were significantly different than all other groups and t-test with Welch’s correction confirmed that ω-3 fatty acids were preferentially elevated in spaceflight. The ω-3/ω-6 ratio was increased by ~ 26% in spaceflight mouse livers as compared with ground controls. The ω-6/ω-3 ratio in the NASA foodbar given to both groups was 10 [13]. Notably, our metabolomics results show significant increase in docosahexaenoate (DHA; 22:6n3) in spaceflight (log_2_ FLT/AEM = 0.5, *P* = 0.043; Table 3).

## Discussion

### Spaceflight enhances fat accumulation in the liver

Despite adequate intake of energy and protein by astronauts, spaceflight results in catabolism of lean body mass in a flight duration-dependent manner and an increase in body fat [1]; body weight alone does not account for changes in energy balance. Our DXA measurements showing a 22% increase in the fat to body weight ratio in FLT mice as compared with ground controls are consistent with human spaceflight and bed rest studies, as is the apparent atrophy of muscle mass [33]. We measured an increase in total hepatic triglycerides; more lipid droplets were visualized using CARS and Oil Red O staining and genes involved in triglyceride biosynthesis were upregulated in FLT mice as compared with AEM controls. Packaging the lipids into droplets may protect the mitochondria from lipotoxicity when β-oxidation is overloaded or impaired and expression of genes involved in lipid droplet formation (*Plin3* and *Plin4*) and growth (*Cidec*) were indeed elevated. However, expression levels of genes involved in lipid droplet lipolysis (*Plin5*; [34–36]), triglyceride lipolysis (*Pnpla2*; *Atgl*) and β-oxidation (*Cpt1a*) were also upregulated. We postulate that lipid turnover is increased in the livers of FLT mice, with enhanced “anabolic” mechanisms such as fatty acid transport offsetting “catabolic” mechanisms such as lipolysis, resulting in a net retention of lipids. Although decreased choline has been linked with fatty liver [37], it is more likely that the relative dehydration of the FLT mice led to the increased production of the osmolyte betaine at the expense of choline and related metabolites, contributing to the dynamic metabolism of the glycerophospholipid membranes surrounding the lipid droplets.

### Spaceflight may induce initiation of HSC activation

We sought to determine whether compositional differences existed between lipid droplets in livers of FLT mice *vs*. AEM controls. We used spontaneous confocal Raman spectroscopy and found notable chemical differences between FLT and AEM mice at 1593 cm^-1^ in the Raman spectra. This component had a negligible effect on the CH band; it did not affect the 3010 cm^-1^ peak (= C-H) and the unsaturation ratio (I3010/I2850) did not change between AEM and FLT mice. A strong candidate for this chemical component is the highly conjugated dietary fatty acid retinol (Vitamin A), present in the NASA foodbar given to both groups [12, 13, 38]. Retinol is almost exclusively stored in HSC lipid droplets and is released when HSCs are initially activated [39–41]. The significant increase in *Lepr* mRNA expression, a marker for HSC activation and differentiation [42], combined with the loss of retinoids, suggests nascent HSC activation occurs in the FLT mice. HSC lipid droplets can either be smaller droplets bounded by a membrane or larger, membrane-free droplets [43] and reduced droplet size correlates with HSC activation and loss of retinoids [44]. The most marked differences in the retinol peak occurred in the largest droplets (AEM livers with the largest droplets had the most retinol signal) and livers of AEM mice had more HSCs containing membrane-free lipid droplets, again supporting the potential for early-stage HSC activation. Remarkably, altered retinol levels and concomitant fatty liver was observed in rats subjected to 14 days of hindlimb suspension, the “gold standard” ground-based spaceflight analog for microgravity [45].

Activated HSCs transform to proliferating myofibroblasts, secreting collagen and remodeling the ECM. We did not see increased mRNA expression of *Acta2* and collagen genes, which are late stage markers of HSC activation. Immunohistochemistry of ACTA2 also did not show differences between the groups. Notably, PPARγ expression was significantly increased. PPARγ expression is negatively correlated with ACTA2 [46], which may potentially explain why we observed loss of retinol without a concomitant increase of ACTA2. Since HSCs are only a small percentage of total hepatic cellular content, it is also possible that changes in expression of some markers in HSCs are masked by expression levels in other cell types. We did, however, observe upregulation in expression of *Serpine1* and *Tgfbr* subunits and downregulation of *Mmp2*, markers of ECM remodeling.

We did not observe significant changes in the number of collagen fibrils between the groups by Picrosirius Red staining. Interestingly, more collagen fibrils are observed in FLT mice livers using SHG imaging; this technique may be more sensitive to different fibril types and more work needs to be done to optimize measurement of these nascent fibrils. Increase in fibrosis in rodents occurs following an extended period of insult [47, 48] and ~13 days of exposure to microgravity is unlikely to induce pathology, particularly for rodents where hepatic pathologic outcomes are not as severe as those observed in humans [47]. However, it is possible that we are observing the genesis of liver insult and that significantly longer duration exposure to stressors in space will result in increases in some of these markers. Interestingly, expression of pro-fibrotic genes was increased in mouse lung tissue after similar duration spaceflight, and histology showed pro-fibrotic changes as well [49], supporting the notion of short-duration spaceflight initiating a nascent fibrogenic response.

### Retinoid metabolism and DHA biosynthesis may modulate HSC activation

Following export from HSCs, protein-bound retinoids may be transported systemically, taken up by other cells, and metabolized to bioactive isoforms (as reviewed [30]. Hormonal retinoids, such as all-trans or 9-cis retinoic acid (atRA and 9cRA, respectively), regulate expression of target genes *via* activation of retinoic acid receptors (RAR) and retinoid X receptors (RXR) [50]. RXR heterodimerizes with RAR, PPARs, FXR and liver X receptors (LXR), nuclear receptors responsible for regulating glucose and lipid metabolism. We mapped our transcriptomics data onto the KEGG pathway for retinol metabolism to attempt to elucidate the fate of retinoids that may be exported from HSCs and retained in the liver. As summarized in Table 2, mRNA expression of the PPARα target *Retsat* was highly upregulated in the FLT mice. Retsat catalyzes the production of all-trans-13,14-dihydroretinol (DROL), a ligand for RARs and a weak analog of atRA [51]. Additionally, *Cyp26a1* expression was significantly downregulated. CYP26, an RAR target, specifically hydroxylates atRA, converting it to isomers with little affinity for nuclear receptors, and also has a role in clearing bioactive retinoids [52]. Taken together, the transcriptomics data suggest upregulation of atRA deactivation pathways [53], a potentially protective response to elevated bioactive retinoid levels following HSC activation. These data did not show highly significant upregulation of genes involved in the metabolism of retinol to 9cRA, a putative RXR ligand.

Supporting the notion of a protective or compensatory response to HSC activation is the increased concentration of hepatic DHA we observed, which is correlated with retinol loss [54]. DHA and arachidonic acid bind RXRα and are activating ligands [9] and increased RXRα expression inhibits HSC activation and suppresses markers of fibrosis in rats [55]. The combination of PPAR, RAR and RXR agonists was shown to be protective against activation in rat HSCs [56]. Taken together, our results suggest that DHA biosynthetic pathways may be upregulated in spaceflight concomitant with retinol loss as a compensatory response to protect HSCs from activation, or to maintain homeostasis. In some studies, retinoids induce hypertriglyceridemia and hepatic lipogenesis whereas in others, excess atRA leads to increased β-oxidation [57]. Although protective deactivation pathways may be upregulated, exposure to spaceflight appears to paradoxically induce both hypertriglyceridemia and increased β-oxidation, which may be a protective response to lipotoxicity. It will be important in the future to work out mechanistic details of these interactions using ground-based models such as hind-limb unloading.

### PPARα-mediated pathways are activated in livers of FLT mice

PPARα, fatty acids and retinoids regulate an overlapping set of target genes that impact a variety of functions including fatty acid uptake and intracellular binding, mitochondrial β-oxidation and peroxisomal fatty acid oxidation, ketogenesis, triglyceride turnover and bile synthesis/secretion [58, 59] and PPARα expression was significantly upregulated in FLT mice. ACOTs are thioesterases (and PPARα targets) that play important roles in fatty acid metabolism, engaging in hydrolase, carboxyesterase and lipase activity [60, 61]. ACOT activity may control the pool of NEFAs transported to the nucleus available for activating PPARα [62] which regulates their transcription [63], suggesting existence of a self-maintaining loop allowing modulation of PPAR signaling [64].

Cytoplasmic ACOT1, together with CYP4, mediates increased ω-oxidation and the generation of dicarboxylic acids [63], a rescue pathway when β-oxidation is impaired or overloaded [65] and oxidative stress is increased. We observed up-regulation of *Cyp4a31* mRNA expression levels and significant increases in products of ω-oxidation, supporting activation of this PPARα-modulated pathway in spaceflight. Localized to the mitochondria [66], ACOT2 activity is linked to increased fatty acid oxidation, as well as elevated ketogenesis, levels of NEFAs and liver triglycerides [63]. Increased expression of *Acot2* and β-hydroxybutyrate without concomitant upregulation of canonical ketogenesis genes in FLT mice support a spaceflight-induced increase in fatty acid oxidation as well as the potential for a more direct role of ACOT2 in ketogenesis that may be regulated by PPARα.

**Fig 7:**
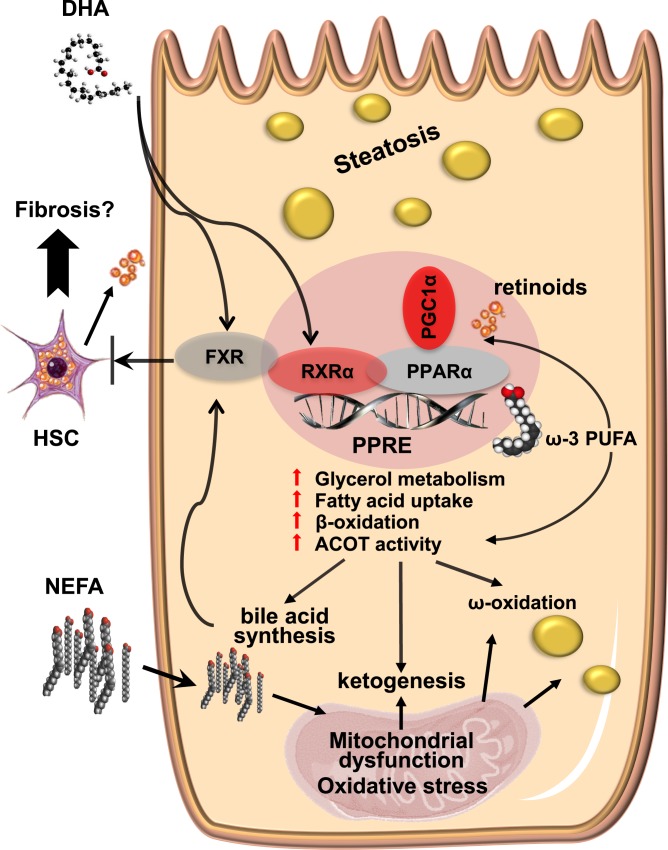
Spaceflight induces activation of PPARα pathways maintained by a feedback loop involving hepatic thioesterase activity and mediated by DHA. Elements of the space environment such as microgravity, oxidative stress and radiation may lead to activation of the PPARα-RXRα heterodimer by ω-3 fatty acids (including DHA), PGC-1α and retinoids from activated HSCs, increasing thioesterase activity. Hepatic steatosis, as well as synthesis of bile acids, ketone bodies and dicarboxylic acids, results from activation of downstream pathways. Fibrosis may also ensue from transformation of the activated HSCs. DHA and bile acids are ligands for FXR, which may be activated in a compensatory manner and help protect from HSC-induced remodeling of the ECM. PPRE, peroxisome proliferator response element.

ACOT8 is a type II thioesterase localized to the peroxisome; over-expression may inhibit peroxisomal β-oxidation to such an extent that hepatic lipids accumulate in droplets [66]. ACOT8 mediates the generation of free bile acids formed from metabolism of cholesterol [63]; we not only observed increased cholate but also a hydrophobic shift in the composition of the bile acid pool (increased cholate and decreased glycocholate) due to decreased *Baat* expression [67]. Increased bile acid hydrophobicity may result in hepatic alterations and tissue injury [68]. Notably, both cholate and DHA are FXR activating ligands [69] and increased *Fgf21* expression is a marker for FXR activation [70]. FXR and bile acids have emerged as important regulators of liver metabolism, affecting lipid and glucose homeostasis as well as hepatic inflammation (as recently reviewed [69, 71, 72]) and FXR activation protects murine livers from HSC activation and fibrosis [73]. Although canonical FXR signaling does not appear to be activated (*Shp* and *Cyp7A1* expression levels are not upregulated), our data suggest the potential for cross-talk between PPAR and FXR pathways and spaceflight-induced activation mediated by DHA, as modeled in Fig 7. More long-term studies, with comprehensive analyses of total bile acid pools, are needed to determine whether increased exposure to the space environment will result in frank hepatic injury.

## Conclusions

We have demonstrated that spaceflight results in increased hepatic triglyceride storage along with a loss of retinoids from HSC lipid droplets. Mining data from multi-‘omics experiments helped to explain these observations and develop the hypothesis that spaceflight, *via* HSC and thioesterase activation, induces and maintains PPARα-mediated pathways, leading to early NAFLD. Although the data are preliminary, we are intrigued by the possibility of spaceflight-mediated modulation of FXR signaling and its potential compensatory role in protection from nascent HSC activation. A notable caveat of this study is that these mice were returned to Earth prior to tissue harvest, therefore the stress of re-entry and acclimatization must be considered when evaluating these data. Mobilization of NEFAs from adipocytes can result from a response to stress. On the other hand, a state of chronic stress, particularly when energy is not used, such as in the spaceflight environment, can result in a hormonal shift, altering metabolic homeostasis [74]. Mice have not yet been sampled in microgravity; it is possible they suffer from chronic stress during spaceflight, however it is possible that exit and reentry are the stressors. The adult liver is a primary regulator of lipid homeostasis, and as such it carries out multiple metabolic processes; the observations described here will clearly influence development of necessary countermeasures to protect astronaut health on long-duration spaceflights such as a mission to Mars (or for individuals on Earth confined to bedrest for long periods of time). Further study in this area is merited and analysis of tissues harvested in space from mice flown aboard the ISS for several months may help determine whether long-term spaceflight might lead to hepatic injury and whether damage can be prevented.

## Supporting Information

S1 Fig A dominant peak corresponding to a Raman shift near 1593 cm^-1^ is observed for retinol. A Raman spectrum of a pure retinol standard shows the presence of a major peak at 1593 cm^-1^. The high wavenumber region of the spectrum (inset) is also markedly different than that obtained in tissue samples.
(PDF)

S2 Fig Pathway analysis of FXR signaling. IPA analysis was performed using no fold cutoff and P = 0.1 on the entire gene set. Pathways for cholesterol secretion were activated, as well as those promoting hepatic bile accretion. Red indicates upregulated expression; green indicates downregulated expression in FLT mice as compared with AEM control animals. The intensity of color correlates with magnitude of up or downregulation.
(PDF)

S3 Fig Concentration heat map of fatty acids. Concentrations for fatty acids identified by metabolomics are displayed as a heat map for *n* = 6 mice per group. Green shading indicates a negative value for log_2_ FLT/AEM concentration values (down-regulated), while red indicates a positive value (up-regulated). The most notable differences between groups are in the ω-6 and ω-3 PUFAs, particularly DHA, DPA and EPA.
(PDF)

S1 File Supplemental methods. (DOCX)

S1 Table Pump wavelengths corresponding to the Raman shifts of interest for CARS imaging, and SRS hyperspectral interrogation at the C-H stretching band and at the higher frequency region of the fingerprint band. (DOCX)

S2 Table Biochemicals identified in spaceflight (FLT) and ground control (AEM) livers. (XLSX)

S3 Table Raman shifts and associated molecular vibrations. (DOCX)

S4 Table Genes, pathways and processes associated with metabolites upregulated in spaceflight. (DOCX)

## Abbreviations

ACOT: Acyl-coA thioesterase
AEM: Animal enclosure module
AtRA: All-trans retinoic acid
BAT: Brown adipose tissue
CARS: Coherent ant-Stokes Raman spectroscopy
DHA: Docosahexaenoate
DXA: Dual-energy X-ray absorptiometry
ECM: extracellular matrix
EGAN: Exploratory gene association networks
FLT: Flight
FXR: Farnesoid X receptor
GO: Gene ontology
HSC: Hepatic stellate cell
KEGG: Kyoto encyclopedia of genes and genomes
NAFLD: Non-alcoholic fatty liver disease
NEFA: Non-esterified fatty acid
PPAR: Peroxisome proliferator-activated receptor
RAR: Retinoic acid receptor
RXR: Retinoid X receptor
SRS: Stimulated Raman scattering
STS: Space transportation system
