# Correction: Spaceflight Activates Lipotoxic Pathways in Mouse Liver

**DOI:** 10.1371/journal.pone.0155282

**Published:** 2016-05-04

**Authors:** Karen R. Jonscher, Alba Alfonso-Garcia, Jeffrey L. Suhalim, David J. Orlicky, Eric O. Potma, Virginia L. Ferguson, Mary L. Bouxsein, Ted A. Bateman, Louis S. Stodieck, Moshe Levi, Jacob E. Friedman, Daila S. Gridley, Michael J. Pecaut

The images for Figs 3 and 4 are incorrectly switched. The image that appears as Fig 3 should be Fig 4, and the image that appears as Fig 4 should be Fig 3. The figure captions appear in the correct order. Please see the corrected Figs 3 and 4 here.

**Fig 3:**
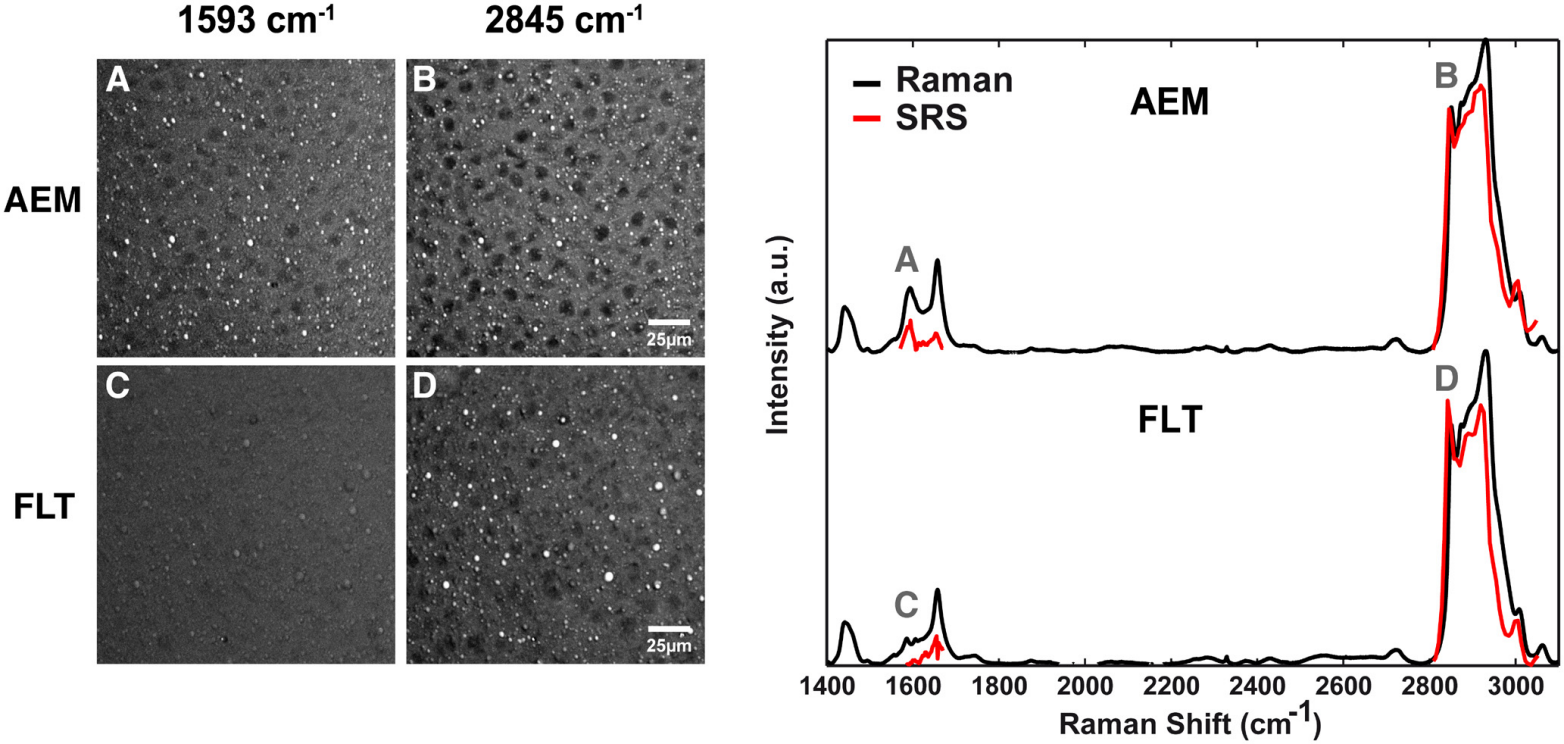
Lipid droplets in spaceflight mouse liver have reduced retinol content. Stimulated Raman scattering (SRS) images of liver sections (left panel) revealed a decreased intensity of embedded lipid droplets at ~1593 cm^-1^ for spaceflight mice (FLT, *C*) with respect to ground mice (AEM, *A*), and showed no difference at 2845 cm^-1^ (*B*, *D*), the C-H_2_ symmetric stretching band characteristic of lipids. Hyperspectral SRS imaging (right panel) around the two frequencies of interest unveil quasi-identical spectra of the lipid droplets of the two mouse groups, except for the peak at ~1593 cm^-1^ (red curves). Spontaneous Raman spectra (black curves) agree with the SRS results. Raman spectra were acquired using *n* = 3 mice per group, 2 sections per mouse, and 2–3 droplets per section. Technical replicates were also performed for several samples. SRS imaging was used to confirm the Raman results and was performed on sections from one mouse in each group.

**Fig 4:**
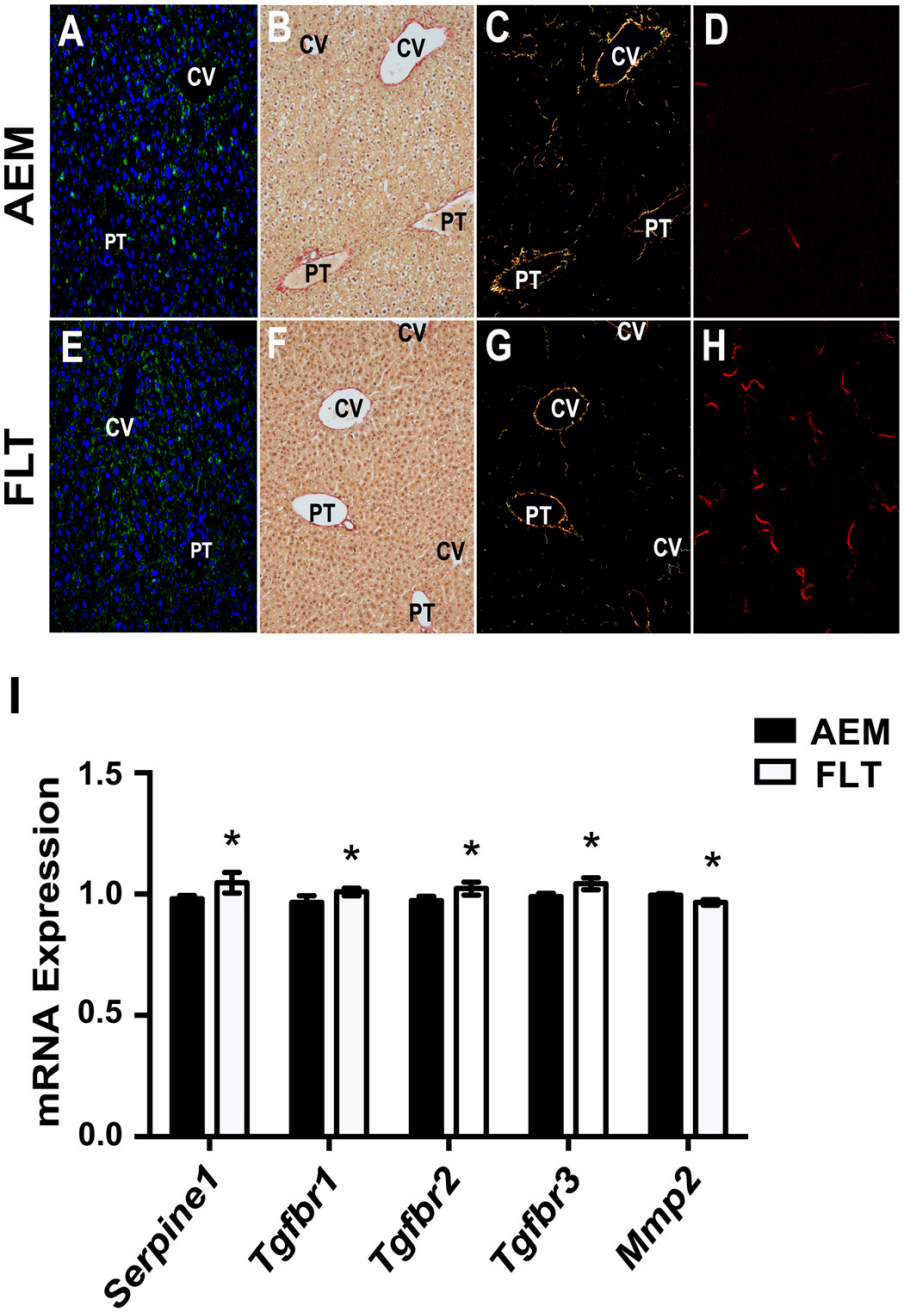
Remodeling of the ECM is increased in livers from spaceflight mice. Fixed tissues were stained for PLIN2 (green) and ACTA2 (red) and nuclei were stained with DAPI (blue) to visualize lipid droplets and markers of activation in stellate cells (*A*, *E*). Mouse liver tissues were either fixed as described in Materials and Methods and stained with Picrosirius Red (*B*, *C*, *F*, *G*) or flash frozen and cryosectioned (*D*, *H*). Collagen staining was observed in Picrosirius Red stained sections (*B*, *F*) primarily near the portal triad (PT) and central vein (CV) areas. Using cross-polarized light, signal was more readily observed (*C*, *G*). SHG imaging was performed on cryosectioned samples (*D*, *H*) and increased collagen signal in interstitial regions was observed in livers from FLT mice as compared with AEM controls. Representative images are shown from each group (*n* = 3–5 mice/group). Magnification is 200× for stained samples and 60× for SHG images. Transcriptomic analysis reveals that expression levels of several important modulators of ECM remodeling are changed in spaceflight (*I*). Values are mean ± SEM; * P < 0.05. *n* = 5–6 / group.

Additionally, there was an error in S1 Fig, where an older version was inadvertently included. Please see the corrected S1 Fig here.

## Supporting Information

S1 Fig A dominant peak corresponding to a Raman shift near 1593 cm^-1^ is observed for retinol. A Raman spectrum of a pure retinol standard shows the presence of a major peak at 1593 cm^-1^. The high wavenumber region of the spectrum (inset) is also markedly different than that obtained in tissue samples.
(PDF)

## References

[1] Jonscher KR, Alfonso-Garcia A, Suhalim JL, Orlicky DJ, Potma EO, Ferguson VL, et al (2016) Spaceflight Activates Lipotoxic Pathways in Mouse Liver. PLoS ONE 11(4): e0152877 doi: 10.1371/journal.pone.0152877 27097220 10.1371/journal.pone.0152877 PMC4838331

