# Supplementary figures and images for: Spaceflight Activates Lipotoxic Pathways in Mouse Liver
Source: PLoS One. 2016 Apr 20;11(4):e0152877. doi: 10.1371/journal.pone.0152877 (PMC4838331; doi:10.1371/journal.pone.0152877)

A

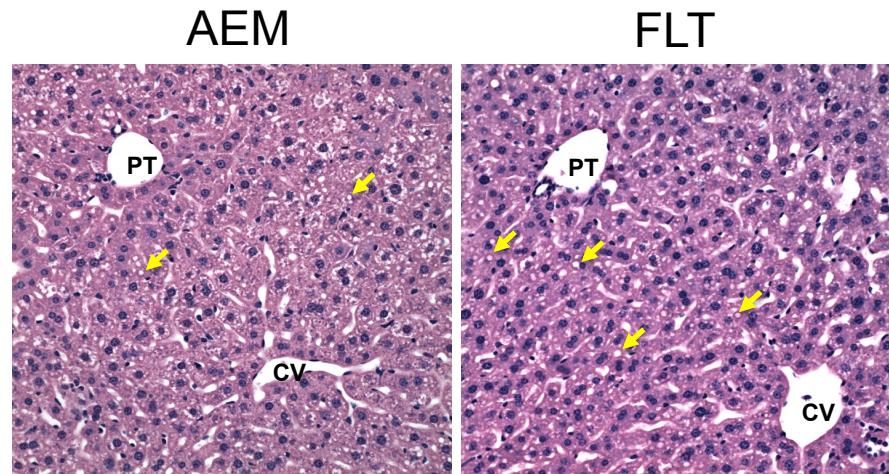

B

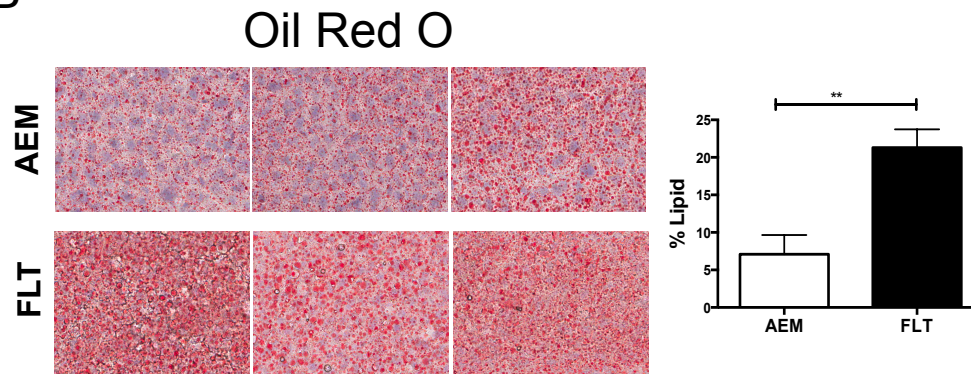

Supplement: S1 Fig — A Raman spectrum of a pure retinol standard shows the presence of a major peak at 1593 cm-1. The high wavenumber region of the spectrum (inset) is also markedly different than that obtained in tissue samples. (PDF) [file pone.0152877.s001.pdf]

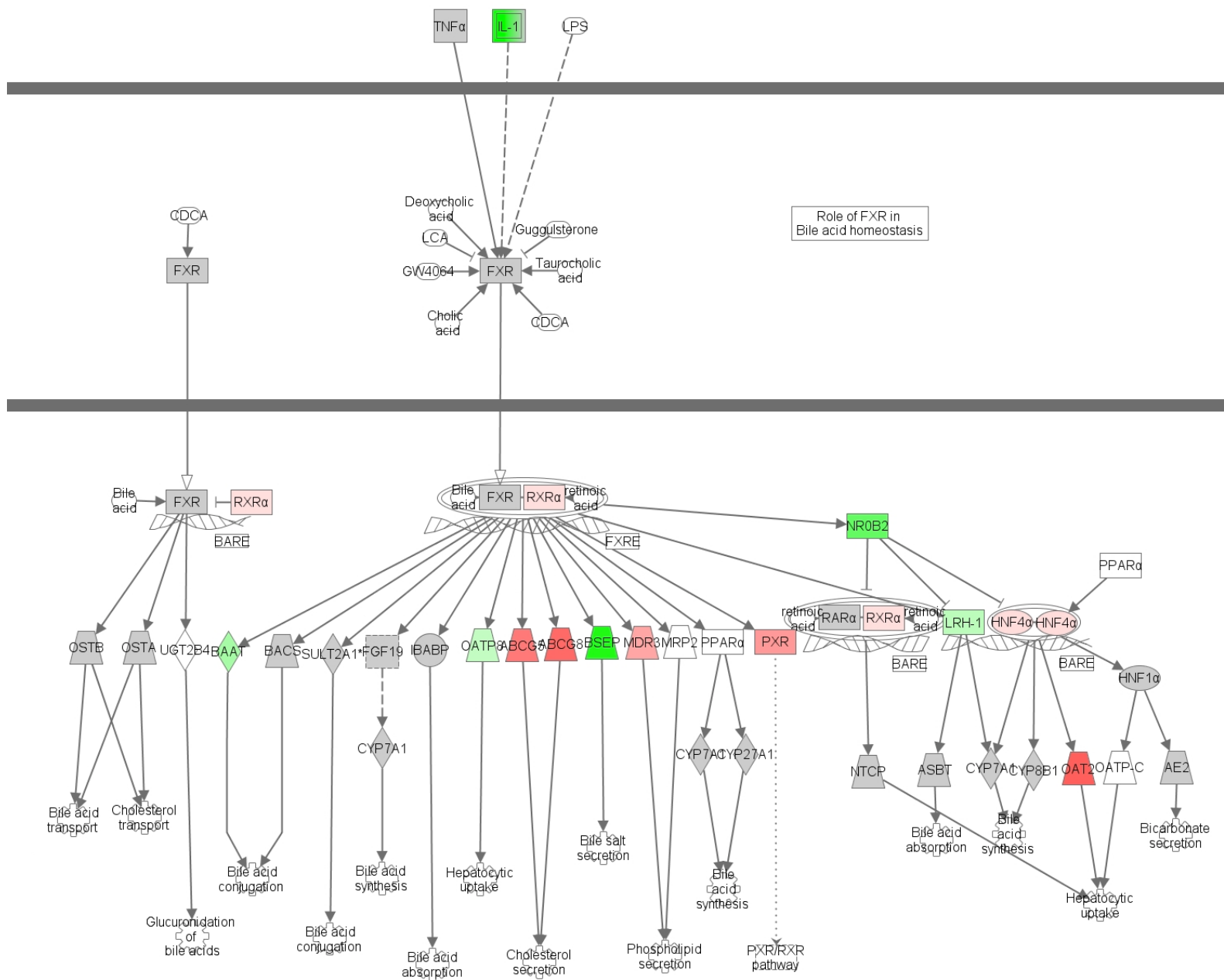

Supplement: S2 Fig — IPA analysis was performed using no fold cutoff and P = 0.1 on the entire gene set. Pathways for cholesterol secretion were activated, as well as those promoting hepatic bile accretion. Red indicates upregulated expression; green indicates downregulated expression in FLT mice as compared with AEM control animals. The intensity of color correlates with magnitude of up or downregulation. (PDF) [file pone.0152877.s002.pdf]
