# Supplementary material for: Spaceflight Activates Lipotoxic Pathways in Mouse Liver
Source: PLoS One. 2016 Apr 20;11(4):e0152877. doi: 10.1371/journal.pone.0152877 (PMC4838331; doi:10.1371/journal.pone.0152877)
Supplement: S3 Fig — Concentrations for fatty acids identified by metabolomics are displayed as a heat map for n = 6 mice per group. Green shading indicates a negative value for log2 FLT/AEM concentration values (down-regulated), while red indicates a positive value (up-regulated). The most notable differences between groups are in the ω-6 and ω-3 PUFAs, particularly DHA, DPA and EPA. (PDF) [file pone.0152877.s003.pdf]

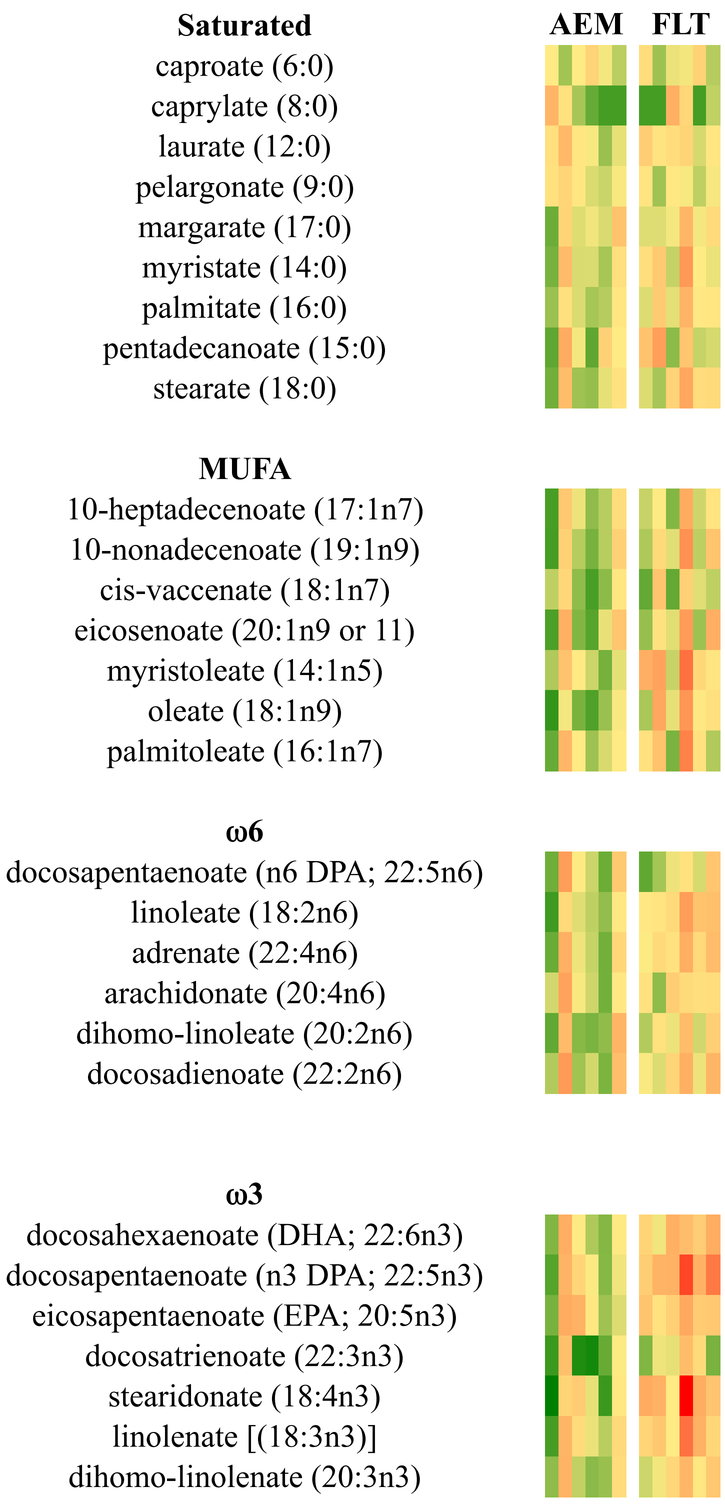
AEM
FLT
Saturated
caproate (6:0)
caprylate (8:0)
laurate (12:0)
pelargonate (9:0)
margarate (17:0)
myristate (14:0)
palmitate (16:0)
pentadecanoate (15:0)
stearate (18:0)
MUFA
10-heptadecenoate (17:1n7)
10-nonadecenoate (19:1n9)
cis-vaccenate (18:1n7)
eicosenoate (20:1n9 or 11)
myristoleate (14:1n5)
oleate (18:1n9)
palmitoleate (16:1n7)
ω6
docosapentaenoate (n6 DPA; 22:5n6)
linoleate (18:2n6)
adrenate (22:4n6)
arachidonate (20:4n6)
dihomo-linoleate (20:2n6)
docosadienoate (22:2n6)
ω3
docosahexaenoate (DHA; 22:6n3)
docosapentaenoate (n3 DPA; 22:5n3)
eicosapentaenoate (EPA; 20:5n3)
docosatrienoate (22:3n3)
stearidonate (18:4n3)
linolenate [(18:3n3)]
dihomo-linolenate (20:3n3)
