# Supplementary material for: Spaceflight Activates Lipotoxic Pathways in Mouse Liver
Source: PLoS One. 2016 Apr 20;11(4):e0152877. doi: 10.1371/journal.pone.0152877 (PMC4838331; doi:10.1371/journal.pone.0152877)
Supplement: S1 File — (DOCX) [file pone.0152877.s004.docx]

**Spaceflight activates lipogenic pathways in the liver**

**Short Title: Spaceflight, lipogenesis and liver**

Karen R. Jonscher, Alba Alfonso-Garcia, Jeffrey Suhalim, David J. Orlicky, Eric O. Potma, Mary L. Bouxein, Ted A. Bateman, Virginia L. Ferguson, Louis S. Stodieck, Moshe Levi, Jacob E. Friedman, Daila S. Gridley and Michael J. Pecaut

**Supplemental Methods**

**Target Preparation/Processing for GeneChip Analysis**

A maximum of 30-mg frozen liver tissue was used and all steps were performed at room temperature. Buffers were prepared according to the manufacturer’s instructions. Total RNA was isolated using an RNeasy kit (Qiagen, Chatsworth, CA) and processed as recommended by Affymetrix, Inc. (Affymetrix GeneChip Whole Transcript Sense Target Labeling Assay Manual, Affymetrix, Inc., Santa Clara, CA). Eluted total RNAs were quantified by NanoDrop (ThermoScientific, Wilmington, DE) and the concentrations of sample aliquots were adjusted to 100ng/ul. Total RNA samples were assessed for quality prior to performing target preparation/processing steps by loading approximately 25-250ng of each sample onto a RNA 6000 Nano LabChip and evaluated on an Agilent Biolanalyzer 2100 (Agilent Technologies, Palo Alto, CA).

The Ambion WT expression kit (Life Technologies, Carlsbad, CA) was used to prepare RNA samples for whole transcriptome microarray analysis. Briefly, random hexamers that are tagged with a T7 promoter are used in first strand synthesis of cDNA. Then using the T7 promoter, second strand synthesis is performed and the double stranded cDNA is subsequently used as a template in an *in vitro* transcription reaction to generate many copies of antisense cRNA. 10ug of antisense cRNA is input into a second cycle cDNA reaction using reverse transcriptase and random hexamers to produce single stranded DNA in the sense orientation. The single-stranded DNA is fragmented to an average length of 70 bases and then labeled using a recombinant terminal deoxynucleotidyl transferase (TdT) and an Affymetrix proprietary DNA labeling reagent that is covalently linked to biotin. 2ug of the labeled, fragmented single-stranded cDNA is hybridized at 45°C with rotation for 17 hours (Affymetrix GeneChip Hybridization Oven 640) to probe sets present on an Affymetrix GeneChip 1.0ST array. The GeneChip arrays were washed and then stained with streptavidin-phycoerythrin on an Affymetrix Fluidics Station 450 (Fluidics protocol FS450_007). Arrays were scanned using GeneChip Scanner 3000 7G and Command Console Software v. 3.2.3 to produce .CEL intensity files.

Probe cell intensity files (*.CEL) were analyzed in Affymetrix Expression Console software v1.1.1 using the PLIER algorithm to generate probe level summarization files (*.CHP). The settings used were algorithm-PLIER v2.0; quantification scale-Linear; quantification type-signal and detection p-value; background-PM-GCBG; normalization method-sketch-quantile.

Data were subsequently processed with CARMAweb (Comprehensive R based Microarray Analysis web service) using their significance analysis of microarrays (SAM) method to obtain fold changes and p values for determining differential expression. CARMAweb is a free, online tool hosted by Medical University Innsbruck (Rainer 2006 Nucleic Acids Res. 2006 Jul 1;34(Web Server issue):W498-503.). Results were imported into Ingenuity Pathway Analysis (IPA) software (Ingenuity Systems, Inc.) where we characterized changes in gene expression pathways related to lipid metabolism.

**Raman Spectroscopy:** Coherent anti-Stokes Raman scattering (CARS) and Stimulated Raman scattering (SRS) signals were obtained by combining two laser beams: a Stokes beam fixed at 1064 nm, and a pump beam tuned to the wavelength of interest (Supplemental Table 1). The system consisted of an optical parametrical oscillator (OPO; Levante, Emerald OPO, Berlin, Germany) pumped by a 76 MHz mode-locked Nd:Vanadate laser (Picotrain, High-Q, Hohenems, Austria) that delivered a fundamental beam at 1064 nm (Stokes beam) with 7 ps pulses, and a second harmonic generated beam at 532 nm. The latter pumped the OPO that generated the tunable beam that served as the pump beam in the CARS process. Spectral tuning of the pump beam was possible by adjusting the crystal temperature, the Lyot filter, and the cavity length of the OPO with custom-written computer code. The two beams were overlapped both temporally and spatially, and sent into a laser scanner (Fluoview 300, Olympus, Center Valley, PA), attached to an inverted microscope (IX71, Olympus). The combined beams were then focused through a 20×, 0.75 NA objective lens (UplanS Apo, Olympus) onto the sample. CARS signal was collected by a photomultiplier tube (R3896 PMT; Hamamatsu, Hamamatsu City, Japan) after passing a 625 filter with a 95 nm bandwidth (Thorlabs, Newton, NJ).

SRS images were obtained by detecting the stimulated Raman loss of the pump beam. For this purpose, the Stokes beam was modulated at 10 MHz with an acousto-optic modulator (AOM; Crystal Technology, Palo Alto, CA). The modulation of the pump intensity was detected by a photodiode (FDS1010; Thorlabs, Newton, NJ), and the signal was demodulated with a home-built lock-in amplifier. The average combined power of Stokes and pump beams at the specimen was kept under 50 mW throughout this study to avoid sample degradation. Additional spontaneous Raman spectra from the mouse liver were acquired with a commercial Raman microscope (InVia Confocal; Renishaw, Wotton-under-Edge, Gloucestershire, UK). Supplemental Table 3 summarizes relevant vibrational modes and their molecular assignments. Multiple droplets and multiple sections were analyzed from *n* = 4 mice per group.

**Second Harmonic Generation (SHG) and Two-Photon Autofluorescence (TPAF) Microscopy**

SHG and TPAF microscopy were used for label-free collagen imaging. Cryosections (16 um thick) on microscope slides were rinsed with cold PBS to remove OCT and coverslipped. Images were acquired at 60× using a Zeiss 510 microscope (Carl Zeiss, Jena, Germany) equipped with a Coherent Chameleon Ultra II laser (Coherent, Santa Clara, CA). The average laser power at 800 nm (tuned for SHG) at the laser aperture was approximately 4W with 140 fs pulse duration and 80 MHz repetition rate. After passing through the microscope optics, the pulse duration was approximately 300 fs. SHG signal was detected on a non-descanned detector (NDD) following transmission through a filter cube containing a narrow band 390 – 410 nm emission filter (hq400/20m-2p, Chroma Technology, Bellows Falls, VT). TPAF signal at 450 – 700 nm was similarly detected following reflection from a dichroic mirror at 425 nm (hq575/250m-2p, Chroma Technology, Bellows Falls, VT). Sections from *n* = 3 mice per group were scanned in 2-3 regions of interest. Images were captured using Zen software (Zeiss).
