# Supplementary material for: Spaceflight Activates Lipotoxic Pathways in Mouse Liver
Source: PLoS One. 2016 Apr 20;11(4):e0152877. doi: 10.1371/journal.pone.0152877 (PMC4838331; doi:10.1371/journal.pone.0152877)
Supplement: S1 Table — (DOCX) [file pone.0152877.s005.docx]

**S1 Table.** Pump wavelengths corresponding to the Raman shifts of interest for CARS imaging, and SRS hyperspectral interrogation at the C-H stretching band and at the higher frequency region of the fingerprint band.

|  | **λ_pump_ (nm)** | **Raman Shift (cm^-1^)** |
| --- | --- | --- |
| CARS | 817 | 2845 |
| SRS:CH | 820-803 | 2800-3050 |
| SRS: Fingerprint | 912-904 | 1565-1665 |
