# Supplementary material for: Spaceflight Activates Lipotoxic Pathways in Mouse Liver
Source: PLoS One. 2016 Apr 20;11(4):e0152877. doi: 10.1371/journal.pone.0152877 (PMC4838331; doi:10.1371/journal.pone.0152877)
Supplement: S3 Table — (DOCX) [file pone.0152877.s007.docx]

**S3 Table.** Raman shifts and associated molecular vibrations

| **Raman shift**  **(cm^-1^)** | | **Molecular vibration** |  |
| --- | --- | --- | --- |
| **1440** | Lipid/protein, CH deformation | | |
| **1593*** | Retinol, conjugated C=C stretch | | |
| **1660** | Lipids, C=C stretching band | | |
| **2845** | Lipids, C-H_2_ symmetric stretch | | |
| **2885** | Lipids, C-H_2_ stretch | | |
| **2950** | Lipid/protein, C-H_3_ stretch | | |
| **3010** | Lipid, =C-H stretches | | |
