# Supplementary material for: Spaceflight Activates Lipotoxic Pathways in Mouse Liver
Source: PLoS One. 2016 Apr 20;11(4):e0152877. doi: 10.1371/journal.pone.0152877 (PMC4838331; doi:10.1371/journal.pone.0152877)
Supplement: S4 Table — (DOCX) [file pone.0152877.s008.docx]

**S4 Table.** Genes, pathways and processes associated with metabolites upregulated in spaceflight.

| **Entrez Gene** | **Gene**  **Name** | **FLT/AEM** | ***P* value** | **KEGG** | **Pathway Name** | **Visible Enrichment** | **Gene Ontology** | **Process Name** | **Visible Enrichment** |
| --- | --- | --- | --- | --- | --- | --- | --- | --- | --- |
| 171210 | *Acot2* | 2.01 | 1.71E-08 | 1100 | Metabolic pathways | 2.69E-79 | GO:0006629 | lipid metabolic process | 4.84E-79 |
| 14555 | *Gpd1* | 1.61 | 1.05E-06 | 4975 | Fat digestion and absorption | 3.70E-36 | GO:0044255 | cellular lipid metabolic process | 1.51E-63 |
| 66853 | *Pnpla2* | 1.44 | 1.83E-08 | 564 | Glycerophospholipid metabolism | 1.53E-28 | GO:0043436 | oxoacid metabolic process | 6.65E-56 |
| 14571 | *Gpd2* | 1.43 | 2.81E-10 | 590 | Arachidonic acid metabolism | 1.07E-27 | GO:0019752 | carboxylic acid metabolic process | 6.65E-56 |
| 71911 | *Bdh1* | 1.10 | 5.45E-06 | 592 | alpha-Linolenic acid metabolism | 2.82E-27 | GO:0006082 | organic acid metabolic process | 1.98E-55 |
| 19016 | *Pparg* | 0.95 | 9.71E-04 | 4972 | Pancreatic secretion | 1.76E-26 | GO:0042180 | cellular ketone metabolic process | 3.65E-55 |
| 12908 | *Crat* | 0.94 | 7.49E-08 | 591 | Linoleic acid metabolism | 4.75E-24 | GO:0016042 | lipid catabolic process | 4.31E-52 |
| 16891 | *Lipg* | 0.87 | 3.02E-07 | 565 | Ether lipid metabolism | 2.71E-23 | GO:0032787 | monocarboxylic acid metabolic process | 5.07E-45 |
| 170789 | *Acot8* | 0.85 | 9.47E-08 | 4730 | Long-term depression | 3.14E-21 | GO:0006631 | fatty acid metabolic process | 5.44E-41 |
| 57279 | *Slc25a20* | 0.81 | 3.16E-07 | 4912 | GnRH signaling pathway | 2.59E-19 | GO:0055114 | oxidation-reduction process | 2.53E-37 |
| 12894 | *Cpt1a* | 0.77 | 6.98E-08 | 561 | Glycerolipid metabolism | 4.51E-19 | GO:0044242 | cellular lipid catabolic process | 1.17E-34 |
| 108114 | *Slc22a7* | 0.76 | 1.75E-03 | 4370 | VEGF signaling pathway | 6.19E-19 | GO:0044106 | cellular amine metabolic process | 4.28E-29 |
| 23945 | *Mgll* | 0.74 | 7.81E-08 | 4664 | Fc epsilon RI signaling pathway | 1.19E-18 | GO:0009308 | amine metabolic process | 3.09E-28 |
| 107045 | *Lars* | 0.71 | 2.60E-07 | 5145 | Toxoplasmosis | 8.37E-18 | GO:0044283 | small molecule biosynthetic process | 5.53E-26 |
| 13434 | *Trdmt1* | 0.69 | 8.71E-05 | 4270 | Vascular smooth muscle contraction | 1.40E-17 | GO:0019637 | organophosphate metabolic process | 1.12E-24 |
| 26457 | *Slc27a1* | 0.69 | 2.35E-05 | 3320 | PPAR signaling pathway | 7.48E-15 | GO:0046486 | glycerolipid metabolic process | 1.02E-23 |
| 69836 | *Pla2g12b* | 0.63 | 5.00E-04 | 4010 | MAPK signaling pathway | 1.66E-13 | GO:0016053 | organic acid biosynthetic process | 4.40E-23 |
| 171282 | *Acot4* | 0.52 | 3.10E-04 | 4260 | Cardiac muscle contraction | 5.17E-13 | GO:0046394 | carboxylic acid biosynthetic process | 4.40E-23 |
| 12116 | *Bhmt* | 0.52 | 2.31E-03 | 5010 | Alzheimer's disease | 1.55E-12 | GO:0006066 | alcohol metabolic process | 1.37E-22 |
| 21813 | *Tgfbr2* | 0.47 | 7.73E-04 | 5016 | Huntington's disease | 2.71E-12 | GO:0046942 | carboxylic acid transport | 2.57E-21 |
| 14411 | *Slc6a12* | 0.46 | 2.87E-04 | 330 | Arginine and proline metabolism | 8.32E-11 | GO:0015849 | organic acid transport | 3.10E-21 |
| 54447 | *Asah2* | 0.41 | 7.83E-03 | 190 | Oxidative phosphorylation | 1.20E-10 | GO:0015718 | monocarboxylic acid transport | 4.22E-21 |
| 20810 | *Srm* | 0.38 | 9.62E-03 | 5012 | Parkinson's disease | 1.35E-10 | GO:0008610 | lipid biosynthetic process | 6.99E-21 |
| 11605 | *Gla* | 0.36 | 4.99E-03 | 480 | Glutathione metabolism | 4.04E-09 | GO:0071702 | organic substance transport | 8.01E-21 |
| 109652 | *Acy1* | 0.33 | 2.23E-02 | 4976 | Bile secretion | 2.17E-08 | GO:0018904 | organic ether metabolic process | 8.31E-21 |
| 16890 | *Lipe* | 0.33 | 1.54E-03 | 980 | Metabolism of xenobiotics by cytochrome P450 | 3.84E-08 | GO:0006639 | acylglycerol metabolic process | 3.61E-20 |
| 56424 | *Stub1* | 0.32 | 3.02E-03 | 4146 | Peroxisome | 4.47E-08 | GO:0006638 | neutral lipid metabolic process | 5.01E-20 |
| 64918 | *Bhmt2* | 0.32 | 2.45E-03 | 270 | Cysteine and methionine metabolism | 4.57E-08 | GO:0044282 | small molecule catabolic process | 1.21E-19 |
| 71670 | *Acy3* | 0.31 | 4.94E-02 | 71 | Fatty acid metabolism | 1.30E-07 | GO:0006644 | phospholipid metabolic process | 1.66E-19 |
| 18263 | *Odc1* | 0.29 | 2.55E-02 | 140 | Steroid hormone biosynthesis | 2.35E-07 | GO:0006662 | glycerol ether metabolic process | 3.52E-19 |
| 11484 | *Aspa* | 0.29 | 2.84E-02 | 340 | Histidine metabolism | 6.24E-07 | GO:0006641 | triglyceride metabolic process | 3.74E-19 |
| 11529 | *Adh7* | 0.28 | 1.76E-02 | 260 | Glycine, serine and threonine metabolism | 1.40E-06 | GO:0010876 | lipid localization | 1.41E-18 |
| 70025 | *Acot7* | 0.28 | 9.36E-03 | 600 | Sphingolipid metabolism | 3.01E-06 | GO:0019395 | fatty acid oxidation | 9.07E-18 |
| 18744 | *Pja1* | 0.27 | 6.06E-03 | 1040 | Biosynthesis of unsaturated fatty acids | 3.00E-05 | GO:0006576 | cellular biogenic amine metabolic process | 1.25E-17 |
| 71519 | *Cyp2u1* | 0.27 | 1.52E-02 | 52 | Galactose metabolism | 4.73E-05 | GO:0034440 | lipid oxidation | 1.28E-17 |
| 66902 | *Mtap* | 0.23 | 8.28E-03 | 982 | Drug metabolism - cytochrome P450 | 6.35E-05 | GO:0006633 | fatty acid biosynthetic process | 1.72E-16 |
| 11717 | *Ampd3* | 0.23 | 6.30E-03 | 460 | Cyanoamino acid metabolism | 8.53E-05 | GO:0006869 | lipid transport | 2.56E-16 |
| 208665 | *Akr1d1* | -0.19 | 2.90E-02 | 61 | Fatty acid biosynthesis | 1.19E-04 | GO:0015908 | fatty acid transport | 1.17E-15 |
| 14870 | *Gstp1* | -0.19 | 1.51E-02 | 4960 | Aldosterone-regulated sodium reabsorption | 1.67E-04 | GO:0035601 | protein deacylation | 1.50E-15 |
| 26568 | *Slc27a3* | -0.19 | 2.26E-02 | 2010 | ABC transporters | 1.79E-04 | GO:0016054 | organic acid catabolic process | 6.85E-15 |
| 13120 | *Cyp4b1* | -0.21 | 4.45E-02 | 380 | Tryptophan metabolism | 1.91E-04 | GO:0046395 | carboxylic acid catabolic process | 6.85E-15 |
| 74134 | *Cyp2s1* | -0.22 | 3.15E-02 | 232 | Caffeine metabolism | 2.04E-04 | GO:0030258 | lipid modification | 7.55E-15 |
| 81701 | *Egfl8* | -0.28 | 7.66E-03 | 4142 | Lysosome | 2.46E-04 | GO:0008202 | steroid metabolic process | 1.49E-14 |
| 74205 | *Acsl3* | -0.31 | 1.25E-02 | 430 | Taurine and hypotaurine metabolism | 2.54E-04 | GO:0006520 | cellular amino acid metabolic process | 1.66E-14 |
| 21366 | *Slc6a6* | -0.36 | 1.12E-02 | 72 | Synthesis and degradation of ketone bodies | 3.10E-04 | GO:0006595 | polyamine metabolic process | 2.84E-14 |
| 22436 | *Xdh* | -0.37 | 9.80E-05 | 983 | Drug metabolism - other enzymes | 3.99E-04 | GO:0046503 | glycerolipid catabolic process | 7.86E-14 |
| 15493 | *Hsd3b2* | -0.51 | 4.10E-02 | 4920 | Adipocytokine signaling pathway | 6.06E-04 | GO:0072329 | monocarboxylic acid catabolic process | 8.95E-14 |
| 14104 | *Fasn* | -0.65 | 1.56E-06 | 830 | Retinol metabolism | 9.04E-04 | GO:0042493 | response to drug | 1.20E-13 |
| 27413 | *Abcb11* | -0.72 | 5.21E-05 | 410 | beta-Alanine metabolism | 2.08E-03 | GO:0006476 | protein deacetylation | 1.29E-13 |

FLT/AEM reflects the log_2_-centered fold change and *P* value is generated by processing microarray data using CARMAWeb. Visible Enrichment values are calculated by EGAN on the selected gene list using a one-tailed Fisher’s Exact test.
