# Supplementary figures and images for: Correction: Spaceflight Activates Lipotoxic Pathways in Mouse Liver
Source: PLoS One. 2016 May 4;11(5):e0155282. doi: 10.1371/journal.pone.0155282 (PMC4856369; doi:10.1371/journal.pone.0155282)

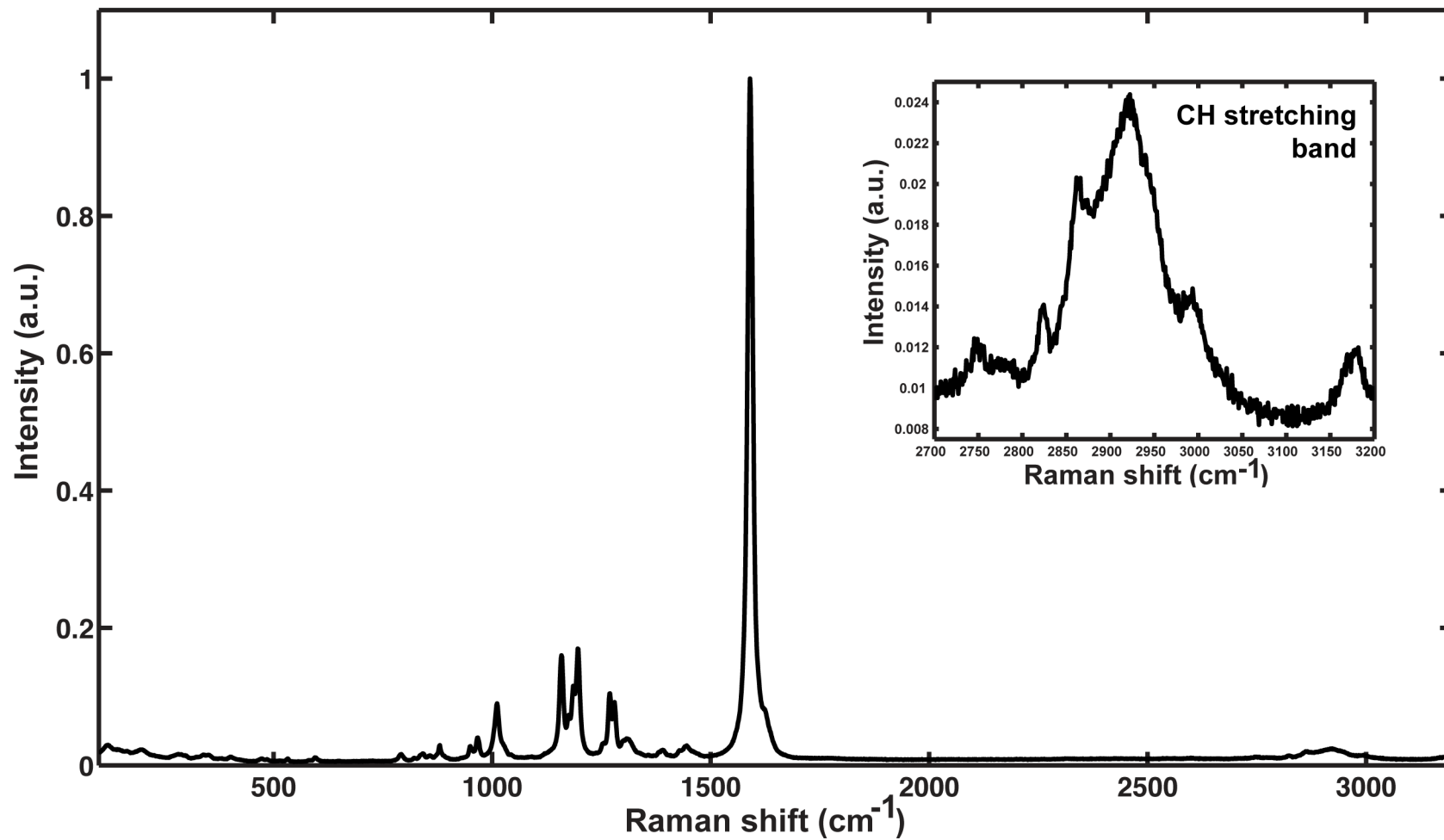

Supplement: S1 Fig — A Raman spectrum of a pure retinol standard shows the presence of a major peak at 1593 cm-1. The high wavenumber region of the spectrum (inset) is also markedly different than that obtained in tissue samples. (PDF) [file pone.0155282.s001.pdf]
